# Dissecting thrombus-directed chemotaxis and random movement in neutrophil near-thrombus motion in flow chambers

**DOI:** 10.1186/s12915-024-01912-2

**Published:** 2024-05-20

**Authors:** Julia-Jessica D. Korobkin, Ekaterina A. Deordieva, Ivan P. Tesakov, Ekaterina-Iva A. Adamanskaya, Anna E. Boldova, Antonina A. Boldyreva, Sofia V. Galkina, Daria P. Lazutova, Alexey A. Martyanov, Vitaly A. Pustovalov, Galina A. Novichkova, Anna Shcherbina, Mikhail A. Panteleev, Anastasia N. Sveshnikova

**Affiliations:** 1 Center for Theoretical Problems of Physico-Chemical Pharmacology, Russian Academy of Sciences, Moscow, Russia; 2 Dmitry Rogachev National Medical Research Center of Pediatric Hematology, Oncology and Immunology, Moscow, Russia; 3 Department of Oncology, Hematology, Immunology, and Rheumatology, University Hospital Tübingen, Tübingen, Germany; 4 Sechenov First Moscow State Medical University, Moscow, Russia; 5 Federal Institute of Technology Zurich, Zurich, Switzerland; 6 Lomonosov Moscow State University, Moscow, Russia

**Keywords:** Chemotaxis, Hemostasis, Neutrophils, Platelets, Thromboinflammation

## Abstract

**Background:**

Thromboinflammation is caused by mutual activation of platelets and neutrophils. The site of thromboinflammation is determined by chemoattracting agents release by endothelium, immune cells, and platelets. Impaired neutrophil chemotaxis contributes to the pathogenesis of Shwachman-Diamond syndrome (SDS). In this hereditary disorder, neutrophils are known to have aberrant chemoattractant-induced F-actin properties. Here, we aim to determine whether neutrophil chemotaxis could be analyzed using our previously developed ex vivo assay of the neutrophils crawling among the growing thrombi.

**Methods:**

Adult and pediatric healthy donors, alongside with pediatric patients with SDS, were recruited for the study. Thrombus formation and granulocyte movement in hirudinated whole blood were visualized by fluorescent microscopy in fibrillar collagen-coated parallel-plate flow chambers. Alternatively, fibrinogen, fibronectin, vWF, or single tumor cells immobilized on coverslips were used. A computational model of chemokine distribution in flow chamber with a virtual neutrophil moving in it was used to analyze the observed data.

**Results:**

The movement of healthy donor neutrophils predominantly occurred in the direction and vicinity of thrombi grown on collagen or around tumor cells. For SDS patients or on coatings other than collagen, the movement was characterized by randomness and significantly reduced velocities. Increase in wall shear rates to 300–500 1/s led to an increase in the proportion of rolling neutrophils. A stochastic algorithm simulating leucocyte chemotaxis movement in the calculated chemoattractant field could reproduce the experimental trajectories of moving neutrophils for 72% of cells.

**Conclusions:**

In samples from healthy donors, but not SDS patients, neutrophils move in the direction of large, chemoattractant-releasing platelet thrombi growing on collagen.

**Supplementary Information:**

The online version contains supplementary material available at 10.1186/s12915-024-01912-2.

## Background

Neutrophils are the predominant polymorphonuclear cells of innate immune system [1], and they are the first cells to be recruited to sites of inflammation [2]. This quick response is facilitated by their ability to detect extracellular chemical gradients and move towards higher concentrations of chemoattractants, such as LTB_4_, CXCL1, IL-8, and others [3]. This process is known as chemotaxis or guided cell migration [4]. Neutrophils are the main participants in the complex interplay between blood coagulation system, immune system, and endothelium, called thromboinflammation, which occurs in diverse pathophysiological situations, such as bacterial infection or cancer [5, 6]. Thromboinflammation is thought to be driven mainly by the interactions between neutrophils and platelets [7]. It is sometimes mixed with immunothrombosis—a process of thrombus formation at the site of immune cell accumulation and activation [8, 9]. Platelets are anucleate fragments of megakaryocytes that circulate in human blood and play critical roles in both hemostasis and immunity [5, 10]. When platelets are activated at the site of injury or inflammation, they release their granules, which contain P-selectin, fibrinogen, von Willebrand factor (VWF), growth factors, and chemoattractants for leukocytes [11–13]. Upon activation, platelets release a combination of chemokines, such as ADP [14] and IL-8 [15]; alternatively, another chemokine, thrombin, is generated on their surface [16]. Thus, neutrophils are rapidly attracted to the site of platelet activation [17].

Earlier, we developed an ex vivo experimental model, which allows observation of thrombus growth, neutrophil adhesion, and movement and platelet-leukocyte interactions [18]. We proposed the following scheme of neutrophil participation in thrombus formation. Granulocytes attach to growing thrombi in a calcium-dependent manner and then descend to collagen through integrin-dependent adhesion, the descended granulocytes collect dead platelets from the growing thrombi, and granulocytes eventually slow down and get arrested. Although the observed behavior of neutrophils in flow chambers is consistent with the phenomenon of chemotaxis, additional studies are needed to verify the possibility of using this experimental setup for the diagnosis of neutrophil motility.

Chemotaxis is known to be disrupted in certain diseases such as Shwachman-Diamond syndrome (SDS) [19]. SDS is a genetic disorder caused by bi-allelic defects in *SBDS* gene and characterized by pancreatic insufficiency and bone marrow failure [20]. Research by Stepanovic et al. [21] demonstrated that the directed movement of neutrophils, but not random movement, is impaired in SDS. Namely, in the absence of chemoattractants, SDS neutrophils moved similarly to those of healthy donors, while, in spatial fMLP gradient, neutrophils from SDS patients demonstrated decreased linear movement velocity, movement persistence, and directional change in response to chemoattractant. SDS neutrophils displayed a chemotaxis (but not random movement) defect due to the altered F-actin polymerization leading to the inefficient orientation capability of neutrophils towards the chemotactic chemical gradient [22].

Here, we show that, in the aforementioned ex vivo model, neutrophils from healthy donors, but not from SDS patients, display preferred localization closer to growing thrombi and position themselves according to the chemokine gradient reconstructed via mathematical modeling. Additionally, an algorithm simulating neutrophil movement could describe experimentally observed neutrophil trajectories in the majority of runs. Together, these findings imply that neutrophil movement in this experimental design is indeed chemotaxis.

## Results

### Ex-vivo analysis of thromboinflammation in Shwachman-Diamond syndrome

**Fig. 1:**
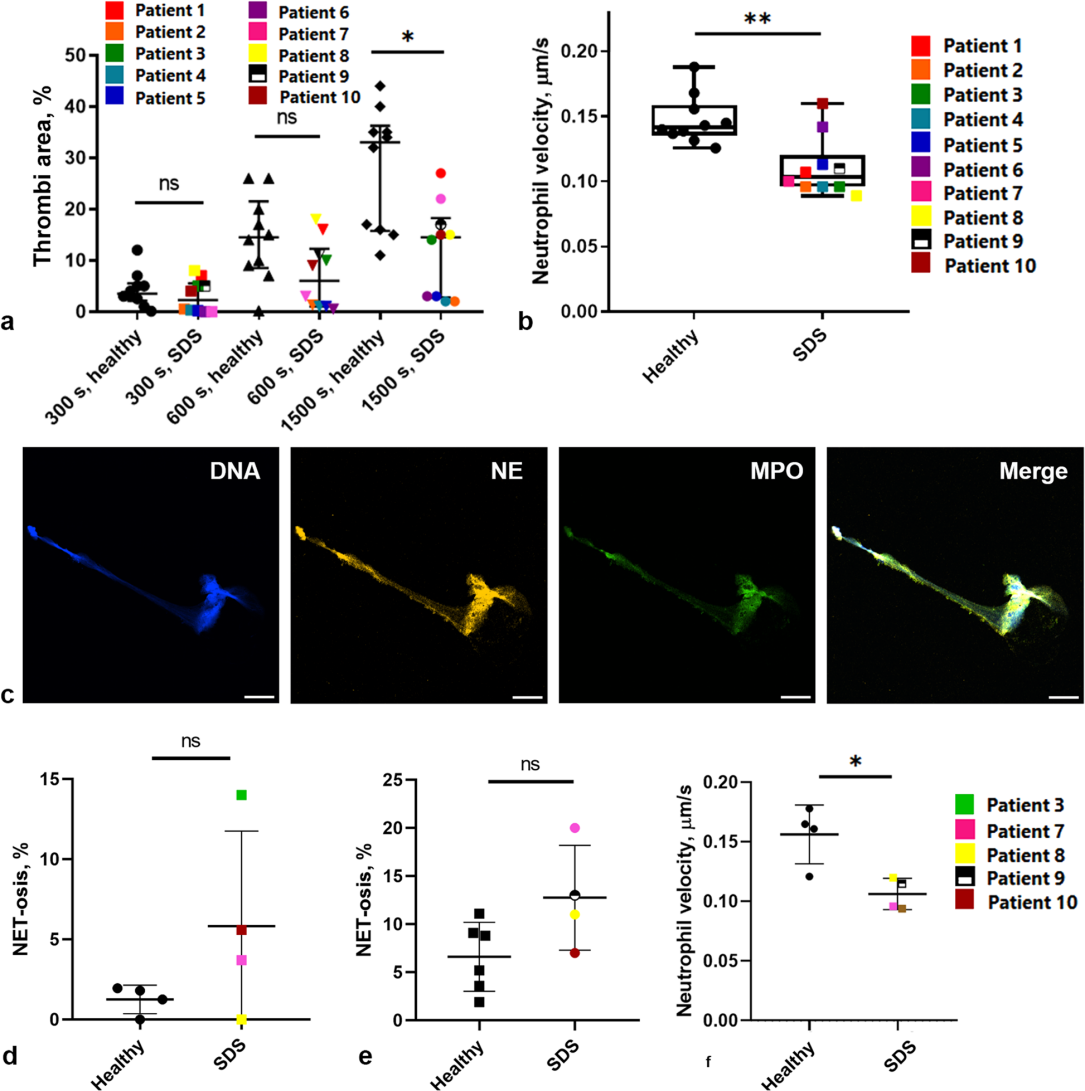
Thromboinflammation parameters and NET-osis levels for SDS patients. **a** Quantification of thrombus growth assay for SDS patients. After 1500 s, SDS patient thrombi are significantly smaller than healthy control thrombi (*n* = 10). **b** Average neutrophil velocities for SDS patients are significantly lower than healthy control neutrophil velocities (*n* = 10). **c** Representative image of SDS patient’s NET; scale bar 10 μm. DNA—staining with Hoechst33342, NE—staining with antibodies against human neutrophil elastase, MPO—staining with antibody against human active myeloperoxidase. **d** NET-osis level in flow chambers (*n* = 4,4). **e** NET-osis level in blood plasma smears (*n* = 6, 4). **f** Average velocities of SDS neutrophils moving around healthy donor’s thrombi compared to average velocities of healthy donor’s neutrophils moving around another healthy donors thrombi (*n* = 4, 4). *p < 0.05; **p < 0.01. Statistical significance was calculated using Mann–Whitney test. Raw data values are given in Additional file 2: Fig. 1

First, we performed analysis [18] of thrombus formation and granulocyte behavior in blood samples from patients with SDS (Fig. 1). It appeared that while thrombus formation was diminished only after 25 min of experiment in SDS samples (Fig. 1a), the neutrophil average velocities were significantly reduced (Fig. 1b) compared to healthy donors. Impaired thrombi growth and/or neutrophil activity in SDS patients could be associated with altered formation of neutrophil extracellular traps (NETs) [23]; that is why we counted NET-s in flow chambers and in leukocyte-rich plasma smears by double-staining with anti-myeloperoxidase (MPO) and anti-neutrophil elastase (NE) antibodies [24] (Fig. 1c). In flow chambers, NET-s (Additional file 1: Fig. S1) appeared rarely both for healthy donors and SDS patients (Fig. 1d). The level of NET-osis measured in blood smears was higher for SDS patients, but without statistical significance (Fig. 1e). Keeping in mind that smaller platelet thrombi could cause lower neutrophil activation and attraction, although clot surface areas did not correlate with neutrophil velocities for SDS patients (Additional file 1: Fig. S2), we performed cross-over experiments, checking SDS patient neutrophil behavior around normal thrombi matrix. When added into flow chamber with healthy donor’s platelets, SDS neutrophils moved significantly slower, than healthy donor’s neutrophils (Fig. 1f). Altogether, these data indicate that the known impairment of neutrophils in SDS could be observed in the proposed ex vivo experimental settings.

### Neutrophils from healthy donors prefer to be situated near thrombi

**Fig. 2:**
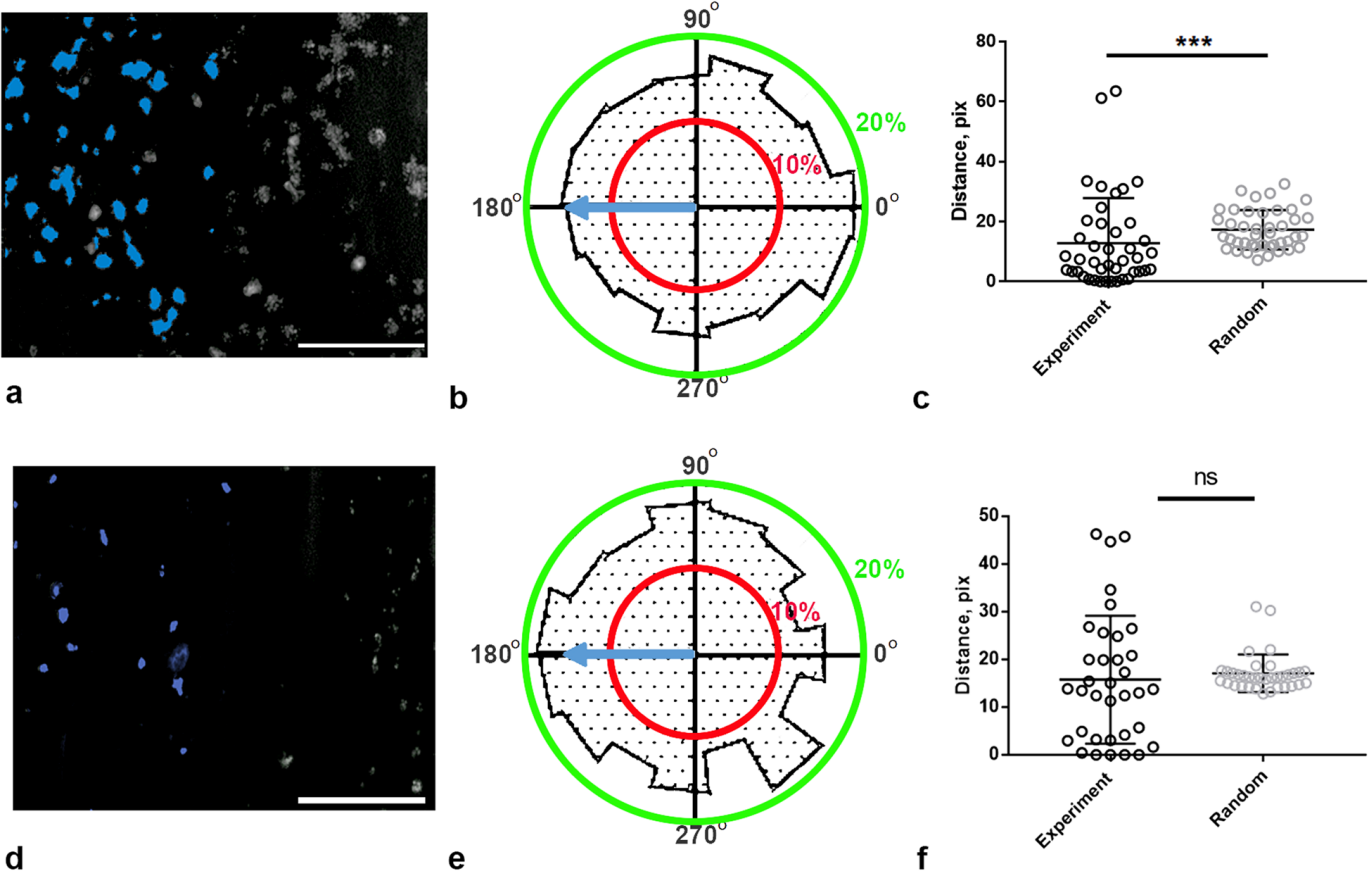
Analysis of experimental neutrophil movement and trajectories for healthy donors and patients with SDS. **a** Representative microscopy image of collagen-induced thrombi growth in a typical healthy donor. Grayscale—DiOC-6, blue—example of thrombi on the half of the image. **b** Histogram of neutrophil movement direction (θ) in samples from healthy donors. Blue arrow indicates flow direction. In healthy donors (*n* = 10, 100 neutrophil trajectories randomly picked from each donor, each trajectory resampled to be at least 250 s long), neutrophils do not predominantly move along the flow. Instead, they appear to favor a counter-flow orientation. **c** The distance from the nearest thrombus edge to the center of neutrophil is notably shorter in experiments compared to the distance from the thrombus border to a randomly placed, neutrophil-sized circle. A representative histogram for one healthy donor. **d** Representative microscopy image of collagen-induced thrombi growth in SDS patient. Grayscale—DiOC-6, blue—example of thrombi on the half of the image. **e** Histogram of neutrophil movement directions in SDS patients. Blue arrow indicates flow direction. **f** In SDS patients, the distance from the nearest thrombus edge to the center of neutrophils is not significantly different from the distance from the platelet border to points of a random trajectory for 8 out of 10 patients. Data for patient #4 is shown. *p < 0.05; **p < 0.01; ***p < 0.001. Statistical significance was calculated using Mann–Whitney test. Raw data values are given in the Additional file 2: Fig. 2

In order to understand what governs neutrophil movement around thrombi, we examined the experimentally obtained videos depicting the movement of neutrophils in the flow chambers (Fig. 2). We measured the angle (θ) between the movement direction and the flow direction (Additional file 1: Fig. S3a) for each video time frame, picking all time frames for randomly selected 60 cells from six different donors. The obtained distribution of directions was shifted against the flow (Fig. 2b); therefore, the flow was not the main factor in the neutrophil movement.

Next, we investigated the spatial locations of neutrophils concerning the position of thrombi. For all motile neutrophils from each donor, the distance (*r*_exp_) between the geometric center of a neutrophil to the nearest thrombus edge were determined (Additional file 1: Fig. S3a) and an average value for each donor was calculated (Fig. 2c). As a control, we generated a random trajectory with the same lengths and velocities as the experimental one starting from a random point on the field of view and calculated the distance (*r*_rand_, Additional file 1: Fig. S3a) from this random trajectory to the closest thrombus’ border. Then, we calculated an average value for a number of independent random trajectories equal to the number of the experimentally observed neutrophils for a particular donor. Remarkably, the trajectories of real neutrophils were significantly closer to thrombi than random trajectories for 8 out of 10 healthy donors (Fig. 2c, Additional file 1: Fig. S4). This pattern suggests that neutrophils tend to position themselves closer to the thrombi. However, an alternative hypothesis suggesting that both platelets and neutrophils might merely adhere to regions with a higher collagen density still needs further investigation.

We performed a similar analysis of the location of SDS neutrophils around thrombi (Fig. 2e,f, Additional file 1: Fig. S5). Interestingly, neutrophils from SDS patients have a tendency to move alongside the flow (Fig. 2e). Only neutrophils from patient #1, who alone has both normal neutrophil and platelet counts, move close to thrombi (Fig. 2f, Additional file 1: Fig. S5). Unlike healthy donors, neutrophils from SDS patients did not show a preference for counter-flow orientation. The lack of counter-flow orientation and of co-localization with thrombi for SDS neutrophils (Additional file 1: Fig. S2) can be attributed to their impaired chemotaxis.

### The roles of blood flow and adhesion proteins in neutrophil behavior

**Fig. 3:**
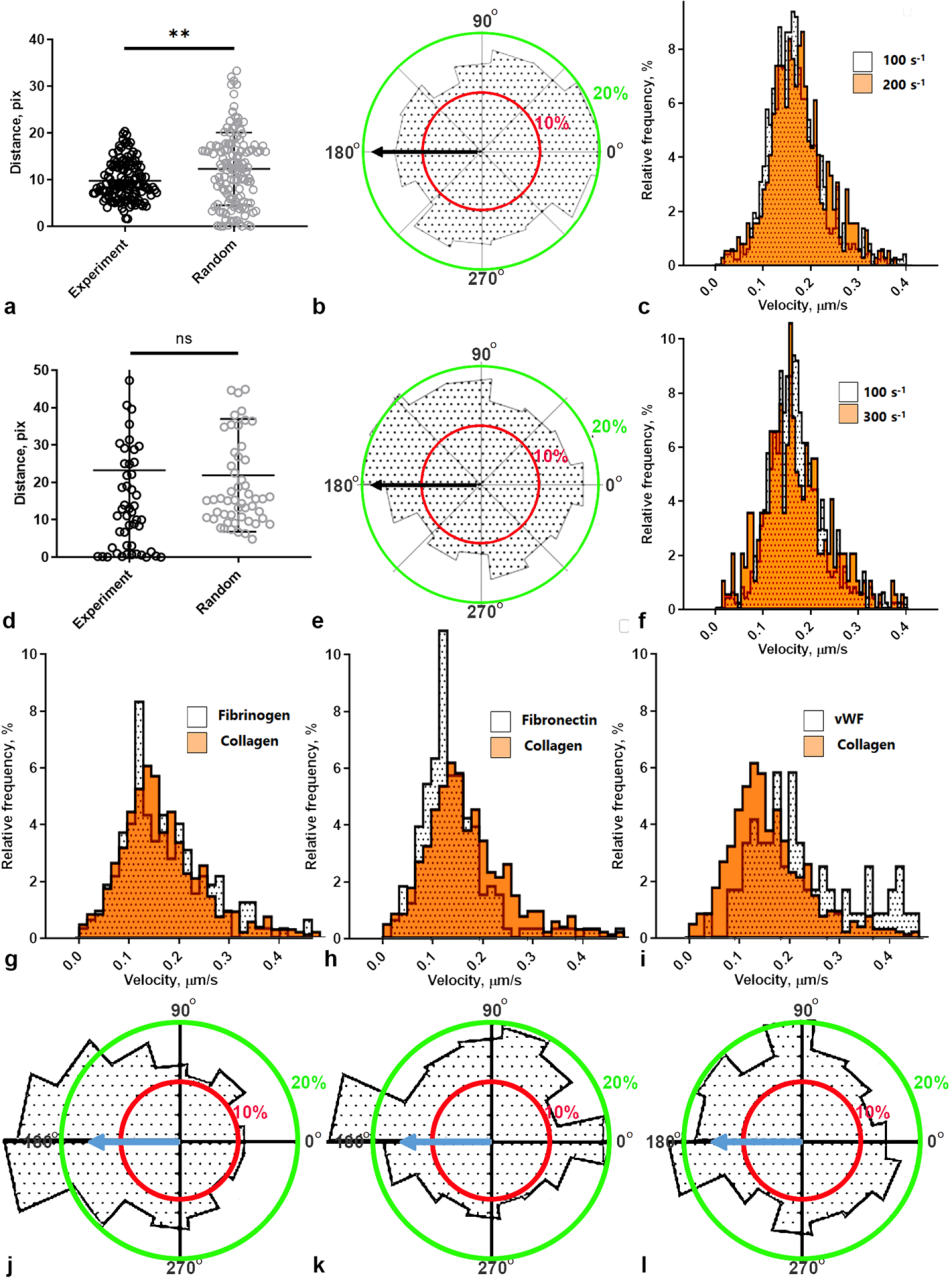
Influence of flow and extracellular matrix proteins presence on neutrophil adhesion, chemotaxis and thrombi growth. **a** The distance from the nearest thrombus edge to the centers of neutrophils is notably shorter for shear stress value of 200 s^−1^ compared to the distance from the thrombi border to random trajectories. **b** Histogram of neutrophil movement directions for *μ* = 200 s^−1^. Blue arrow indicates flow direction. Neutrophils predominantly move counter-flow. **c** Neutrophil velocities distribution for *μ* = 200 s^−1^ and *μ* = 100 s^−1^. No difference appears in neutrophil velocities distribution. **d** The distance from the nearest thrombus edge to the centers of neutrophils is not significantly different from the distance from the platelet border to random trajectories for shear stress value of 300 s^−1^. **e** Histogram of neutrophil movement directions for shear stress value of 300 s^−1^. Blue arrow indicates flow direction. For *μ* = 300 s^−1^, neutrophils predominantly move along the flow. **f** Neutrophil velocities distribution for *μ* = 300 s^−1^ and *μ* = 100 s^−1^. Several velocities’ subpopulations appear for *μ* = 300 s^−1^. *p < 0.05; **p < 0.01; ***p < 0.001. Statistical significance was calculated using Mann–Whitney test. **g**–**i** Neutrophil velocities distribution for *μ* = 200 s^−1^ on fibrinogen (**g**), fibronectin (**h**), or vWF (**i**) covered coverslips. **j**–**l** Histograms of neutrophil movement directions for *μ* = 200 s^−1^ on fibrinogen (**j**), fibronectin (**k**), or vWF (**l**) covered coverslips. Blue arrow indicates flow direction. Neutrophils predominantly move along with the flow. Raw data values are given in the Additional file 2: Fig. 3

In order to investigate the role of the flow (wall shear rate) in the neutrophil movement, we compared neutrophils’ velocities, thrombi area, and amounts of adherent neutrophils for a range of shear rate values, *μ*: 100 s^−1^, 200 s^−1^, 300 s^−1^, 500 s^−1^ (Fig. 3, Additional file 1: Fig. S6, S7). While all observed thromboinflammation parameters were not influenced by the wall shear rate (Additional file 1: Fig. S6, S7), it is notable, that for *μ* = 100 s^−1^, neutrophils preferred to be situated close to thrombi and were predominantly oriented counter-flow (Fig. 3a, b), while for higher shears most of cells rolled with the flow, which was indicated by the appearance of several velocities subpopulations of cells (Fig. 3e, f, Additional file 1: Fig. S7). Two subpopulations with velocities lower and higher than the middle peak could correspond to randomly-moving neutrophils and neutrophils exhibiting rolling with the flow on a significant part of their trajectories, respectively. Interestingly, when the blood flow was stopped after 10 min of perfusion, the average velocities of neutrophils reduced and the counter-flow direction of movement disappeared (Additional file 1: Fig. S8).

Thus, a strong blood flow carries away neutrophils, but it also brings in new platelets and, in theory, creates a chemoattractant gradient that causes neutrophils to move against the flow. To test this hypothesis, we conducted experiments in which thrombus formation occurred on other blood proteins—fibrinogen, fibronectin, and von Willebrand factor (Fig. 3g–l, Additional file 1: Fig. S9). We observed both standard indicators (thrombi area, number and velocities of neutrophils, Additional file 1: Fig. S9) and neutrophil velocity distribution indicators (Fig. 3g–l). Since in all cases the thrombi formed were too small (Additional file 1: Fig. S9), indistinguishable from single adherent platelets, the distances from the cells to the thrombi could not be calculated. None of the used proteins could support counter-flow movement of neutrophils, while thromboinflammation parameters varied for these settings. On fibrinogen, platelet adhesion and neutrophil movement was observed, and linear velocity was not distinguishable from collagen (Fig. 3g, j). The absence of directed movement could be explained by the lack of large platelet aggregates on fibrinogen (Additional file 1: Fig. S9). On vWF coating, no platelet adhesion was observed, and neutrophil movement phenotype was mostly rolling. Result observed on fibronectin, another matrix protein, was similar. Neutrophil and platelet adhesion was observed; however, neutrophil linear velocity was diminished, and neutrophils preferably moved along with the flow.

**Fig. 4:**
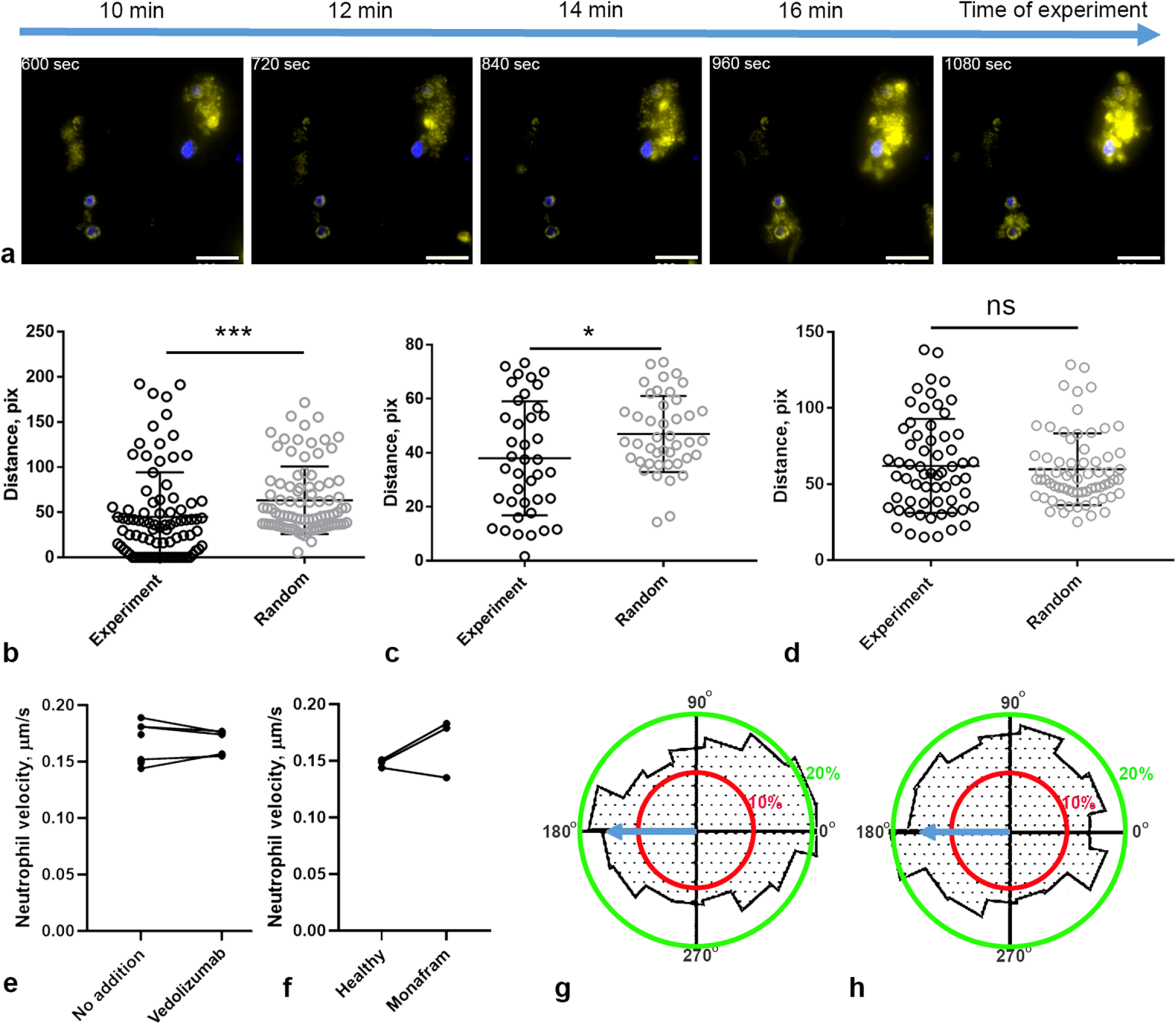
Additional studies of neutrophil adhesion in flow chambers. **a**–**d** Thrombi growth around live tumor cells. **a** A timelapse of a typical thrombus formed around a SiHa cell, blue—Hoechst 33,342 (tumor cells pre-stained), green—DiOC6. Scale bar 30 μm. **b**–**d** The distance from the nearest tumor cell to the centers of neutrophils is notably shorter in this setup compared to the distance from the cell border to randomly placed, neutrophil-sized circles for 4 out of 5 experiments. *p < 0.05; **p < 0.01; ***p < 0.001. Statistical significance was calculated using Mann–Whitney test. **e**–**h** Impact of anti-integrin antibodies Vedolizumab (**e** (*n* = 5), **g**) and Monafram (**f**, **h**) on neutrophil motility upon healthy donor’s blood perfusion with shear rate 200 s^−1^ through flow chamber with collagen-coated coverslip. **e**, **f** (*n* = 3) Average neutrophil velocities. Raw data values are given in Additional file 2: Fig. 4. **g**, **h** Histograms of neutrophil movement directions

The observed tendency of neutrophils to position closer to thrombi can be attributed to the possibility that both platelets and neutrophils might have a tendency for adhering to regions with elevated collagen densities. To eliminate this possibility, we adopted an experimental setup using live oncological cells as foci of thrombus growth and chemoattractants (Fig. 4a). We replicated our previous analysis of neutrophil-to-thrombus distances for this new configuration (Fig. 4b–d). The distance between a neutrophil’s center and the closest thrombus edge was consistently shorter than the distance for randomly placed, neutrophil-sized circles. This reaffirms the inclination of neutrophils to position themselves nearer to thrombi in this setup too, and a parallel-plate flow chamber setup can be used to assess neutrophil chemotaxis.

Another intriguing point is the participation of blood cells and proteins in the observed process. We previously showed that calcium, fibrinogen, and von Willebrand factor are required for neutrophil adhesion, suggesting an interaction through integrins [18]. Here, we continued this research by conducting experiments to observe thromboinflammation in the presence of adhesion inhibitors: Vedolizumab [25] and Monafram [26]. In samples preincubated with Vedolizumab, the number of adhering neutrophils dropped significantly (Additional file 1: Fig. S10a, b), while incubation with Monafram lead to the reduction of thrombi area (Additional file 1: Fig. S9). Both inhibitors had no effect on neutrophil velocity (Fig. 4e, f), indicating that neutrophil motility depends on intrinsic parameters, namely, cytoskeleton proteins, as both in SDS and in Wiskott-Aldrich syndromes [18] the neutrophil movement slows. For Vedolizumab, neutrophil counter-flow orientation persisted (Fig. 4g). However, for Monafram, neutrophil counter-flow directionality was lost (Fig. 4h), indicating disrupted chemotaxis probably due to diminished thrombi size, similar to results obtained for fibrinogen or fibronectin.

Altogether, our data indicate that neutrophils are attracted by the growing thrombi and are influenced by their size. When platelet thrombi are smaller, as on other matrix proteins (Fig. 3, Additional file 1: S9), in presence of inhibitors (Fig. 4, Additional file 1: S10a,b), or with lesser concentrations of collagen, the amount of adherent neutrophils becomes smaller (Additional file 1: Fig. S10c,d). In order to assess the impact of cellular and non-cellular blood components, we performed experiments with total removal of platelets or RBCs or their removal after 10 min of blood perfusion through the flow chamber. In the first case, no thrombi or neutrophils were observed (data not shown). In the second case, neutrophil motility has significantly decreased and the movement directionality was lost (Additional file 1: Fig. S10e-g), indicating that the already grown thrombi stopped to attract neutrophils.

However, there is a question of the different modes of cell motility—chemotaxis, chemokinesis, haptotaxis, and haptokinesis. Chemotaxis is the cell motility guided by soluble chemoattractant gradient, haptotaxis is cell motility guided by chemoattractants adherent to the matrix, and haptokinesis and chemokinesis represent random cell movement [27]. Taking into account the fact that decreasing matrix concentration did not influence neutrophil motility (Additional file 1: Fig. S10), and blood washout significantly decreased neutrophil velocity (Additional file 1: Fig. S10 e–g), we assumed that neutrophils orient in soluble chemoattractant gradient. To be sure, we performed experiments with Asprin, as some NSAIDs such as aspirin influence chemotaxis, but not chemokinesis [28]. Upon 30 min preincubation with 1 mM of Aspirin, neutrophil velocity did not change (Additional file 1: Fig. S10h); however, the counter-flow neutrophil orientation was lost (Additional file 1: Fig. S10i), indicating that neutrophil movement is rather chemotaxis than chemokinesis.

### A theoretical analysis of the chemoattractant distribution and cell movement in flow chambers

**Fig. 5:**
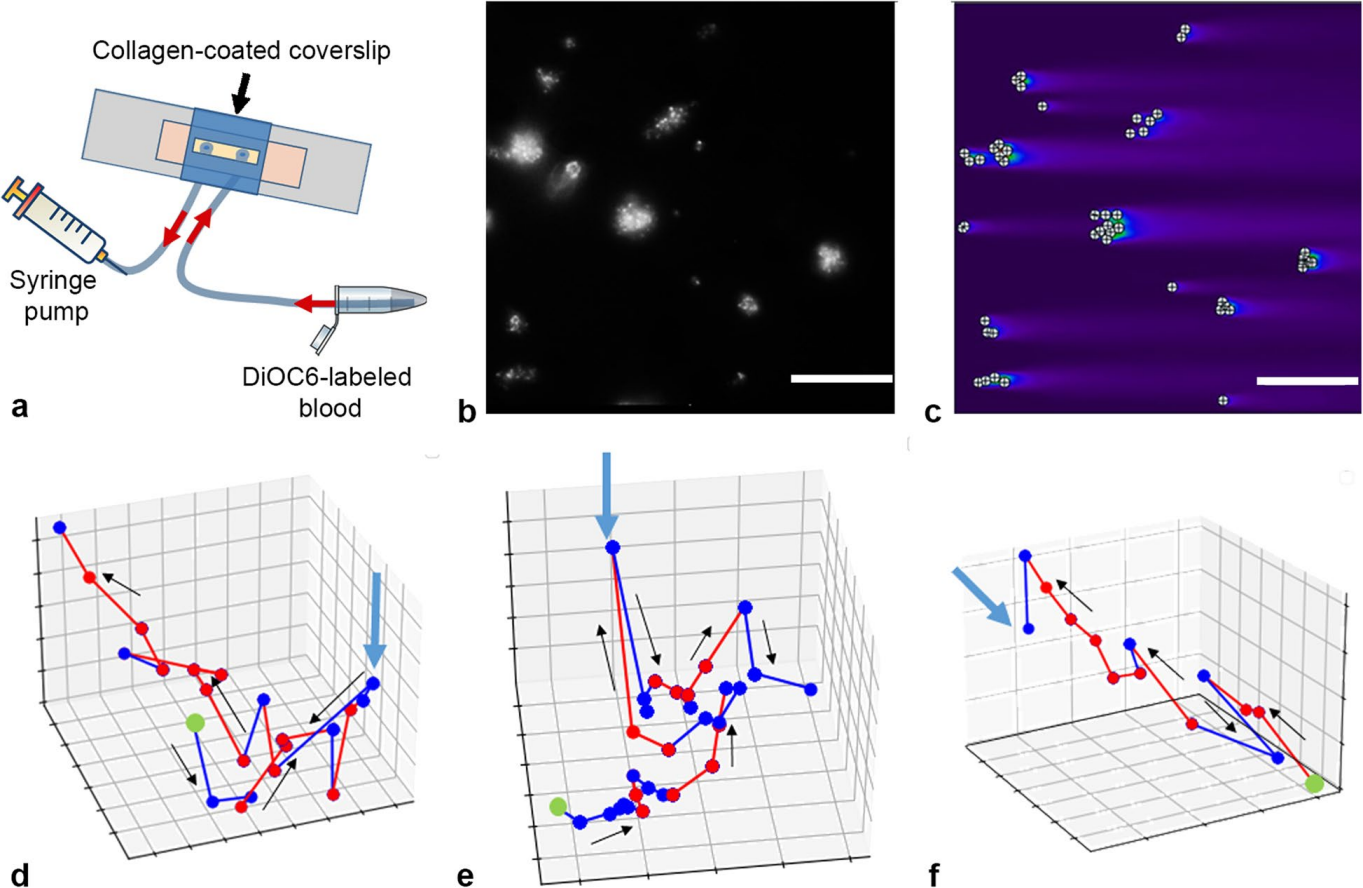
Model chemokine distribution around thrombi in flow chamber. **a** The investigated region in the flow chamber. **b** Experimental data on thrombi location. Scale bar 50 μm. **c** Calculated distribution of a model chemoattractant (CA) around thrombi, depicted in **b**. Long “tails” of CA followed thrombi. **d**–**f** CA concentration along a typical neutrophil trajectory, observed in the experiment. Three typical cells. The start of the trajectory is marked with a green dot. Trajectory segments with elevating chemokine concentrations are denoted with red. Trajectory segments with diminishing chemokine concentrations are denoted with blue. Eventually, some cells wandered into territories with lower chemokine concentrations (blue arrow). Black arrows indicate trajectory directions

The proposition that neutrophils move against the flow due to appearance of a chemoattractant gradient generated by thrombi could be investigated by means of computational modeling. Our results indicate that only growing thrombi generate some chemoattractant, so it could be either CXCL1/2 present in platelets or thrombin generated on their surface; let us name it model chemoattractant (CA). In order to calculate the CA distribution, we have taken distribution of thrombi in a random field of view of a healthy donor (Fig. 1b) and solved the Navier–Stokes equation under the assumption of laminar flow, using a geometry that mirrors our experimental setup to obtain the distribution of flow velocities (Additional file 1: Fig. S3b). We assumed that the thrombi release a chemoattractant (CA) with the following parameters: 8 kDa—molecular mass [29], 2900 molecules per duration of the experiment (30 min)—rate of release [30], 130 μm^2^/s—diffusion rate (taken from experimental data from a cytokine with a similar molecular mass [31]). These parameters were chosen based on the parameters of main platelet-derived chemoattractants—interleukins [32]. Then, we calculated distribution of CA (Fig. 5c). It could be observed that “tails” of CA are distributed behind the thrombi, which explains the preferable up-flow movement of neutrophils, because it means the well-known movement against-gradient [33]. Model computation has been performed for the lowest shear rate of *μ* = 100 s^−1^ due to the fact that for higher shear rates, only a half or less of all donors exhibited definitive chemotaxis. In addition, accurate modeling of neutrophil rolling is out of scope for this model.

**Fig. 6:**
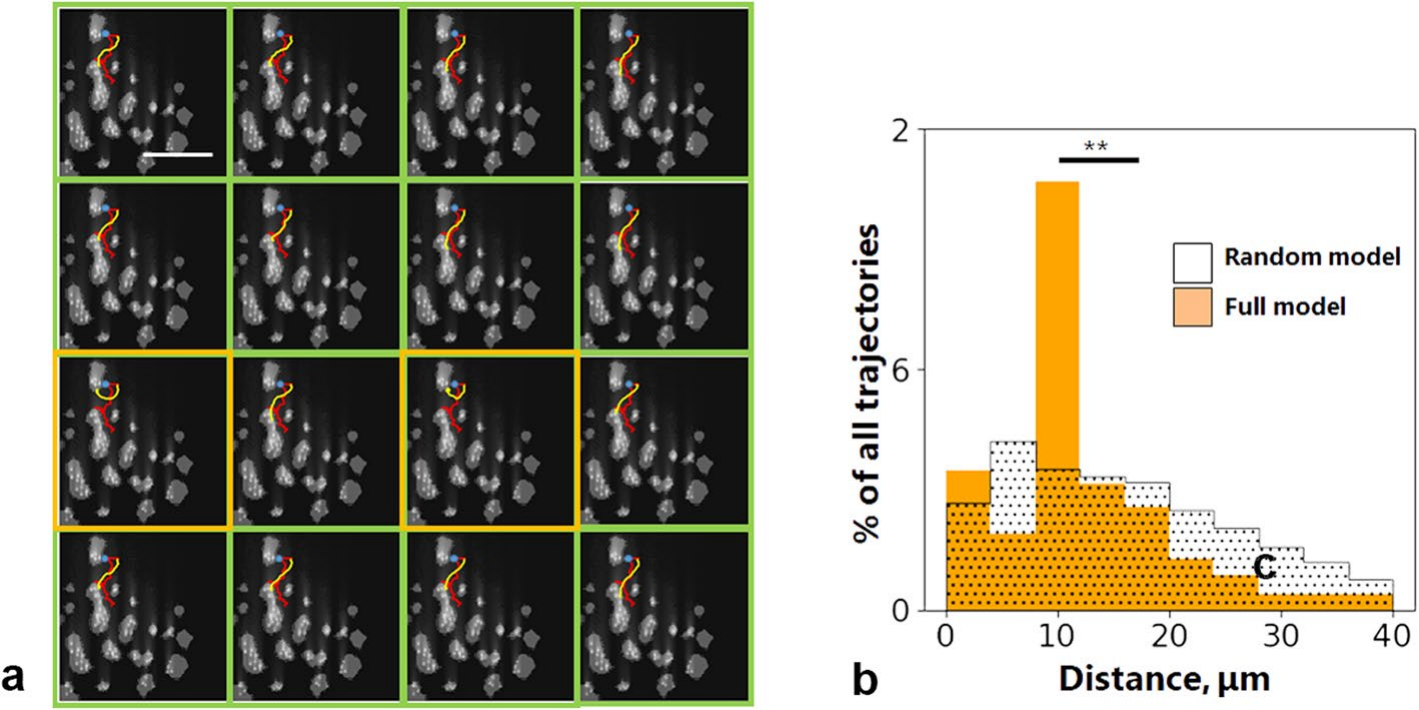
The model of chemotaxis. **a** 16 model runs for a single neutrophil model. Model trajectories successfully describing the experimental neutrophil trajectory are denoted with green frames. Unsuccessful model runs are denoted with orange frames. Scale bare is equal to 40 μm. **b** Distribution of the point-wise distances from the experimental trajectories to the model ones. Orange histogram denotes the full model distances (*N* = 18 cells, 36 trajectories for each cell). Black-and-white histogram denotes the random model distances (*N* = 18 cells, 36 trajectories for each cell). In full model, average distance was 12 μm, compared to 16 μm in the random one. Mann–Whitney non-parametric test was used for comparison. **p < 0.01

Next, we calculated the distribution of CA along the known trajectories of neutrophils (Fig. 5). While neutrophils often moved against CA gradient for 20–40 s, they occasionally ventured into regions with diminishing chemokine concentrations, as highlighted in Fig. 5 (indicated by the blue arrow). Therefore, there are some other factors influencing neutrophil movement. In order to further illustrate this point, we used an algorithm, developed by Szatmary et al. [4], which describes step-wise decision making by a neutrophil, and placed such neutrophil in the chemoattractant field calculated in previous section (Fig. 6). We first selected three neutrophils for each donor, then calculated CA field for a square field of view with a side of 100–250 μm around each neutrophil. Some fields contained more than a single neutrophil. In total, 8 fields were calculated, no less than one for each healthy donor (Additional file 1: Fig. S11). For each neutrophil, we did 36 separate simulations (Additional file 1: Fig. S11, 16/36 trajectories are shown for illustration purposes). The selected neutrophils answered the following criteria: (a) no rolling, (b) no upstream neutrophils within 10 μm (due to LTB_4_ secretion by active neutrophils), (c) the edge of the field of view further than 10 μm (as unknown thrombi might be behind the edge). From 648 trajectories, 433 resembled those of protagonists. But the percentage of successful runs varied greatly between the cells (Additional file 1: Fig. S11). For 5 trajectories out of 18, no model run could describe them; on the other hand, for the other 13, more than 60% runs described them well, and for 8 cells, all runs were successful (Additional file 1: Fig. S11). For a model with CA-insensitive neutrophils, the average point-wise distance from the model trajectory to the experimental one was significantly larger than for the described above model (Fig. 6b). Apart from interleukins, platelets also secrete large amounts of PF4 (CXCL4), which also attracts neutrophils [34, 35]. However, when we assumed the CA to be PF4 (with much lower diffusion coefficient than interleukins), while the absolute concentration of CA increased, the CA gradient did not change, and the model neutrophil moved around the thrombi in the same manner (Additional file 1: Fig. S12).

## Discussion

In this study, we use a combination of microscopy experiments for blood samples from human healthy donors and patients with Shwachman-Diamond syndrome (SDS), in-depth data analysis, and computational modeling to determine the nature of neutrophil movement around growing thrombi in parallel-plate flow chambers. We demonstrate that, in SDS platelets, somewhat smaller thrombi on collagen are formed and neutrophils move more slowly than for healthy donors, while the level of NET-osis is noticeably higher for SDS patients (Fig. 1). While neutrophils from healthy donors are predominantly localized near thrombi and move against the flow, the SDS neutrophils move randomly (Fig. 2). Experiments with adhesion proteins other than collagen (Fig. 3) or integrin inhibitors (Fig. 4) indicated that the counter-flow movement of neutrophils requires large growing thrombi. To explain these findings, we calculated a chemoattractant gradient (Fig. 5), let a model neutrophil [4] move in this field, and compared the resultant trajectory with the real one (Fig. 6).

The necessity of a strong chemoattractant source is supported by the experimental setup where tumor cells served as points of thrombus growth instead of collagen (Fig. 4). Tumor cells are known to promote thrombi growth via molecules on their surface [36] and to attract leukocytes [37], so our results correspond to the literature. On the other hand, in our setting, thrombin is inhibited by hirudin; therefore, the tissue factor pathway, considered to be the main origin of tumor-induced thrombosis [38, 39], could not be responsible for thrombus formation. We assume that, in our setting, the tumor cells attract and activate platelets through either tumor cell podoplanin-platelet CLEC-2 [40, 41] or tumor cell integrin-vWF-platelet GPIb interactions [42]. Therefore, neutrophils could actively participate in the tumor cell invasion process [43]; however, this observation requires further investigation.

**Fig. 7:**
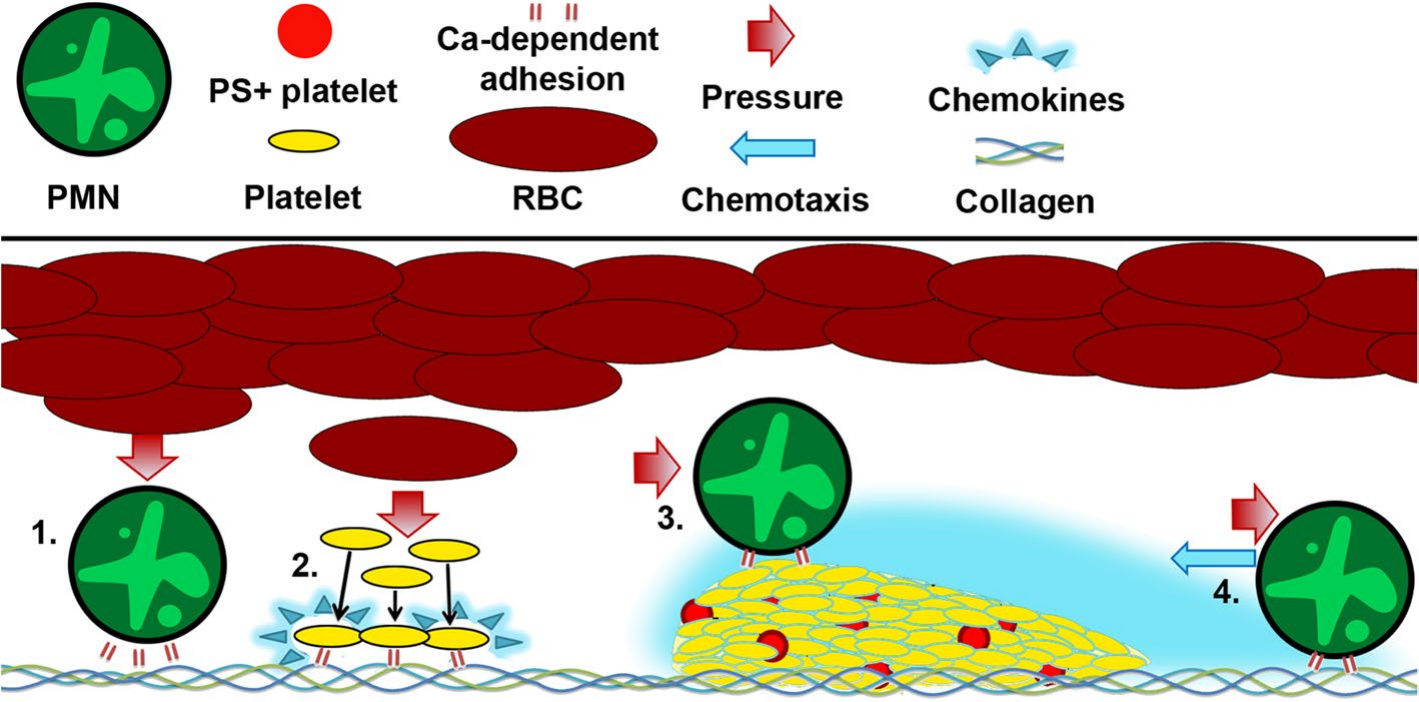
The proposed scheme of granulocytes movement in flow chambers. 1. Red blood cells (RBC) push platelets and leukocytes (PMN) towards collagen-covered surface, where they attach in integrin-dependent manner. 2. When activated, platelets release chemokines (cyan); thus, a growing platelet thrombus constantly releases chemoattractants [3]. 3. When attached to a thrombus, leukocyte moves with the flow. 4. In the chemoattractants, field neutrophil moves counter-flow due to the chemical gradient

Thus, once adhered to the surface, a neutrophil could “sense” the decreasing concentration gradient of the chemokine “tail” trailing a thrombus (Figs. 5 and 7), which would naturally guide a neutrophil to move in a direction opposite to the flow (Fig. 2) [44, 45]. With the increase in flow rates, the number of adherent neutrophils decreases, and the number of rolling ones increases (Fig. 3). These results explain why neutrophils are more important for microthrombi [46] or venous thrombi [47], where flow rates are small enough for their adhesion [48].

Neutrophils from SDS patients do not follow the proposed scheme (Fig. 7). While SDS platelet thrombi successfully grow on collagen (Fig. 1) and other matrix proteins (data not shown), SDS neutrophils move significantly slower even around healthy donors’ thrombi (Fig. 1). The observed random movement of SDS neutrophils (Fig. 2) comes in accordance with the data on disrupted SDS neutrophil chemotaxis [49]. Furthermore, since it has been reported that SDS patients are prone to immune deficiency, the impaired neutrophil chemotaxis alongside neutropenia (Table 1), increased level of NET-osis (Fig. 1), and decreased movement velocities (Fig. 1) can be responsible for the immune dysfunction [50]. Interestingly, for samples from SDS patients, the thrombus area did not correlate with the platelet count, and neutrophil velocities did not correlate with the thrombus area (Additional file 1: Fig. S2), which are interesting observations by themselves because we are not aware of previous reports on hemostasis disorders in SDS.

**Table 1:** Patient characteristics

No	Sex	Age^1^, years	Gene	Mutation	Cytogenetic abnormalities	Platelets^1^, (× 10^9^/L)	ANC^1^, (× 10^9^/L)	rhG-CSF dose, μg/kg per day
1	Male	4	*SBDS*	1. c.258 + 2 T > C 2. c.183_184delinsCT	-	296	6.03	-
2	Male	10	*SBDS*	1. c.258 + 2 T > C 2. c.183_184delinsCT	i(7)(q10)	27	0.59	3.67
3	Female	2	*SBDS*	1. c.258 + 2 T > C 2. c.183_184delinsCT	-	71	23.62	0.43
4	Female	4	*SBDS*	1. c.258 + 2 T > C 2. c.183_184delinsCT	-	156	0.20	1.00
5	Male	2	*SBDS*	1. c.258 + 2 T > C 2. c.183_184delinsCT	-	125	0.86	-
6	Female	7	*SBDS*	1. c.258 + 2 T > C 2. c.183_184delinsCT	-	178	0.61	-
7	Female	8	*SBDS*	1. c.258 + 2 T > C 2. c.183_184delinsCT	-	153	0.74	-
8	Male	10	*SBDS*	1. c.258 + 2 T > C 2. c.183_184delinsCT	-	250	0.21	0.36
9	Female	8	*SBDS*	1. c.258 + 2 T > C 2. c.183_184delinsCT	-	53	1.83	1
10	Female	2	*SBDS*	1. c.258 + 2 T > C 2. c.184A > T	-	161	2.06	-

*Abbreviations*: *ANC* Absolute neutrophil count, *rhG-CSF* Recombinant human granulocyte colony-stimulating factor, *1* At enrollment in the study

Comprehensive computational modeling has long proven itself as an effective approach to unraveling the mechanisms of complex physiological and pathophysiological phenomena [51, 52]. Using a mathematical model of the chemoattractant distribution among the growing thrombi and combining it with a model neutrophil moving in this field, we managed to demonstrate that the observed neutrophil movement was indeed chemotaxis (Fig. 5). However, the model could not describe 28% of cells indicating that other factors should be taken into account. One of the future directions would be to include neutrophil attachment, detachment and rolling into the model.

Further analysis is required to estimate the exact chemoattractant combination emitted by the growing thrombi. Taking into account the turbulence of the blood flow, caused by the forming thrombi, could also improve the obtained results. Finally, following the work of Kaneva et al. [53], stochastic inexplicit incorporation of the physiological mechanisms of the neutrophil chemotaxis, i.e., neutrophil Mac and LFA1 integrin activation [54], could provide an essential improvement to the aforesaid model.

In the proposed experimental setting, several thromboinflammation features could be observed. First, platelet thrombus growth and granulocyte sensitivity to shear flow (Fig. 3). Second, neutrophil directed movement and chemotaxis (Fig. 4). Granulocyte chemotaxis plays a crucial role in various physiological processes such as immune responses, tumor metastasis, wound healing, and the formation of blood vessels [55]. In patients with chemotaxis defects (e.g., harboring inherited mutations in SBDS [21], CXCR4 [56], CXCR2 [57]), the severity of the infection complications could be explained not only by a quantitative deficiency of neutrophils but also by disturbances in the neutrophil movement towards a chemoattractant (for example, towards the focus of inflammation). Consequently, altering chemotaxis is likely to have therapeutic benefits. Having a high-throughput method for conducting granulocyte migration assays is beneficial for assessing and refining potential leads in the field of drug discovery and the optimization of pharmacological screening capability.

## Conclusions

Together with the data obtained in our previous study [18], these results allow us to propose the following scheme of neutrophil involvement in thromboinflammation (Fig. 7). The experiments with other anticoagulants [18], platelets or RBC removal from blood, blood wash-out (Fig. S10), anti-integrin antibodies (Fig. 4, Additional file 1: S10), and variations in wall shear rate (Fig. 3) indicate that RBCs “push” platelets and leukocytes towards the boundaries [58], where they adhere to the surface and to each other through integrins in a calcium-dependent manner. Once activated, platelets release various chemoattractants [29], predominantly interleukins [32]. The necessity of blood plasma demonstrated here (Additional file 1: Fig. S10) indicate that thrombin generated in a growing thrombus also could serve as an attractant. Other adhesion proteins, such as fibrinogen, fibronectin, or VWF (Fig. 3), also support platelet and neutrophil adhesion as well as lower concentrations of collagen (Additional file 1: Fig. S10); however, these proteins do not allow full-fledged thrombus formation and directed neutrophil movement (Fig. 3).

## Methods

### Materials

The sources of the materials were as follows: Annexin V-Alexa Fluor 647 (BioLegend, San Diego, CA), DiOC-6, HEPES, bovine serum albumin, Hoechst-33342, poly-L-lysine, paraformaldehyde (PFA), and phosphate-buffered saline (PBS) (Sigma-Aldrich, St Louis, MO); fibrillar collagen type I (Chrono-Log Corporation; Havertown; USA); Aspirin (Bayer, Germany); Natalizumab (anti-α4β7) (Hospira Inc, USA); Cell Tracker Violet and secondary antibodies were conjugated with AlexaFluor 488 or AlexaFluor 568 (Invitrogen). Human von Willebrand factor (VWF) was a kind gift of Prof. Pierre Mangin (INSERM, Etablissement Français du Sang-Grand Est,UMR_S1255, Fédération de Médecine Translationnelle de Strasbourg, Université de Strasbourg, France), Monafram was a kind gift of Prof. Alexey V. Mazurov (NMRC of Cardiology, Moscow, Russia); human fibrinogen and fibronectin were isolated from human blood plasma and purified by Dr. Egor Osidak (IMTECK, Russia); mouse and rabbit anti-human monoclonal antibodies against myeloperoxidase and neutrophil elastase were a kind gift of Prof. Alexey V. Sokolov (Institute of Experimental Medicine, St. Petersburg, Russia).

### Patients or Human subjects

SDS diagnosis was made based on the typical clinical picture and in all cases confirmed by detection of bi-allelic mutations in the *SBDS* gene.

### Blood collection and handling

Blood was collected into Sarstedt-Monovette© hirudin (525 ATU/ml blood) vacuum tubes. In our previous paper [18], we have shown that heparin increases the percent of immobile neutrophils (probably due to chemotaxis inhibition [59]), and citrate introduces reproducibility issues due to the need of recalcification, without which no neutrophil adhesion was observed. Therefore, we choose hirudin as our primary anticoagulant. Experiments were performed within 3 h after blood collection. For shear stress experiments, blood was collected from adult healthy volunteers (3 female, 3 male donors, *n*_total_ = 6), 22–27 years old. For experiments with different matrix proteins, blood was collected from adult healthy volunteers 18–45 years old (2 female, 2 male donors), *n*_total_ = 4. For experiments with different inhibitors and with blood washout, blood was collected from adult healthy volunteers (5 female, 2 male donors, *n*_total_ = 7), 19–39 years old.

For the assays involving Shwachman-Diamond syndrome patients, blood was collected from healthy pediatric donors (*n* = 5, 2 male, 3 female donors 0.25–33 months old), healthy adult donors (*n* = 5, 3 male, 2 female, 19–29 y.o.) or from patients with Shwachman-Diamond syndrome (Table 1) into Sarstedt-Monovette hirudin (525 ATU/ml blood) tubes. For NET-osis assay in flow chambers, blood was collected either from SDS patients (Table 1) or adult healthy volunteers 18–45 years old into Sarstedt-Monovette hirudin (525 ATU/ml blood). For neutrophil isolation and NET-osis smear preparation, blood from healthy donors and SDS patients was collected into Sarstedt-Monovette EDTA K3E (1.6 mg/ml blood) tubes.

### Patient characteristics

Ten unrelated pediatric patients (6 girls and 4 boys) with Shwachman-Bodian-Diamond syndrome (SDS) were included in this study (Table 1). Median age at enrollment was 5.5 years. Nine patients harbored compound heterozygous mutations in the *SBDS* gene NM_016038.2:c.258 + 2 T > C and NM_016038.2:c.183_184delinsCT. All identified mutations were confirmed by Sanger sequencing. In one patient, isochromosome 7q, i(7)(q10) abnormality was found; all other patients did not have any confirmed cytogenetic abnormalities or signs of MDS/AML. At the time of enrollment, 6 patients received recombinant human granulocyte colony-stimulating factor (rhG-CSF) therapy.

### Fluorescent microscopy

Parallel-plate flow chambers were described previously [18, 60]. Channel parameters were: 0.1 × 18 × 2 mm. Glass coverslips were coated with fibrillar collagen type I (0.2 mg/ml) for 1 h 30 min at 37 °C, washed with distilled water and then inserted into the flow chambers. Alternatively, untreated glass coverslips were used for oncological cell experiments. After addition of fluorescent reagents (DiOC6 (1 μM), Hoechst (2 μg/mL), and AnnexinV-Alexa647 (10 μg/mL)), blood was perfused through the parallel-plate chambers with wall shear rates 0–500 s^−1^ [61]. Thrombus growth and leukocyte crawling were observed in DIC/epifluorescence modes with an upright Nikon Eclipse Ni-U microscope (20x/0.50 Plan Fluor objective) for mathematical model preparation and quantitative assay of experimental neutrophil movement and upright Nikon Eclipse Ni-E (60x/1.49 Apo TIRF objective with oil immersion) for tumor cell experiments.

For blood washout, whole blood was perfused over collagen-coated glass for 10 min and then perfused with either Tyrode’s buffer (137 mM NaCl, 2.7 mM KCl, 12 mM NaHCO3, 0.36 mM NaH2PO4, 1 mM MgCl2, 2 mM CaCl2, 5 mM HEPES (pH 7.5), 0.36% BSA, 1 g/l D-glucose, pH 7.35) with 2 mM calcium or platelet poor plasma (PPP) from the same donor for 5 min at 200 s^−1^. PPP was obtained by centrifuging whole hirudinated blood for 10 min at 1600 g.

To study SDS neutrophil behavior on normal thrombi matrix, EDTA-anticoagulated LRP from either healthy controls or SDS patients was centrifuged at 100 g for 3 min. The pellet was resuspended in 200 μl Tyrode’s buffer and incubated with 1 μM CellTracker Violet fluorescent label for 30 min at 37 °C. The sample was then centrifuged at 100 g for 3 min, the supernatant discarded, and the pellet resuspended in healthy donor whole blood. For healthy controls, whole blood from another donor was used. DiOC6 was then added. Imaging was performed by fluorescent microscopy as described above.

For VWF, fibrinogen, and fibronectin matrix assays, cover glasses were silanized [62] before the experiments. Afterwards, the flow chamber was assembled as described above and incubated for 60 min at 37 °C with either 100 μg/ml vWF, 100 μg/ml fibronectin, or 100 μg/ml fibrinogen. Further assay was carried out as described above.

### NET-osis level determination

For NET-osis observation in a parallel-plate flow chamber, whole blood was perfused over glass coated with 200 μg/ml collagen for 10 min at 100 s^−1^. Non-adherent cells were washed out with Tyrode’s buffer with the addition of 2 mM CaCl_2_. Cells were then incubated for 3 h at 37 °C. Before fixation, the chamber was rinsed with modified Tyrode’s buffer without BSA. Sample fixation was carried out using 1% PFA for 30 min. After rinsing and blocking in 3% BSA in PBS buffer, samples were incubated with primary mouse antibodies against myeloperoxidase (MPO) and rabbit antibodies against elastase for 1 h. Incubation with Hoechst 33,342 and Alexa488- and Alexa555-conjugated secondary antibodies against mouse and rabbit immunoglobulins, respectively, was performed for 1 h.

For smear analysis of NET-osis, leukocyte-rich plasma (LRP) was used [24]. LRP was obtained by sedimenting the blood sample at 37 °C for 45 min. LRP was smeared over a glass microslide and dried overnight at room temperature. The samples were then fixed with 1% formaldehyde for 30 min. After fixation, the smears were washed with 1% BSA and PBS. Subsequently, they were blocked with 10% normal goat serum for 30 min. Thereafter, samples were incubated with primary antibodies to neutrophil elastase and myeloperoxidase for 30 min. Afterwards, samples were incubated with secondary antibodies and Hoechst 33,342 for 20 min. The mounting medium was used to prevent burn-in of fluorescently labeled antibodies. Finally, samples were analyzed by confocal microscopy. The level of NET-osis was determined by the ratio between granulocytes released their DNA and the total number of granulocytes in the sample. Granulocytes were considered as nucleated cells containing MPO and NE that was confirmed with Nikon Ti2 fluorescent microscope with confocal AX attachment.

### Cell culture

The cell lines MCF7 and SiHa (ATCC, USA) were used for our experiments. The cells were cultured as a 2D culture in monolayer in DMEM high glucose (HiMedia, USA) supplemented with 10% FBS (HiMedia, USA) and 100 U/ml penicillin, 100 μg/ml streptomycin solution, and 2 mM L-Glutamine (Sigma-Aldrich, USA). Cells were cultivated at 37 °C in a humidified 5% CO_2_-containing atmosphere. For cells localization in flow chamber, a suspension (10^6^ cells/ml) of cells was incubated in flow chambers with untreated glass for 15 min at 37 °C and then washed with 20 w/v BSA in PBS and afterwards with Tyrode’s buffer (137 mM NaCl, 2.7 mM KCl, 12 mM NaHCO_3_, 0.36 mM NaH_2_PO_4_, 1 mM MgCl_2_, 2 mM CaCl_2_, 5 mM HEPES (pH 7.5), 0.36% BSA, 1 g/l D-glucose, pH 7.35) solution. For exclusive tumor cell staining, Hoechst fluorescent probe was added into the Tyrode’s buffer but not into the whole blood sample.

### Data analysis

The Nikon NIS-Elements software was used for microscope image acquisition; ImageJ (http://imagej.net/ImageJ) was used for image processing. ImageJ manual tracking plugin was used for manual granulocyte tracking.

For automated cell tracking, particle tracking algorithm described in [63] was utilized. The algorithm was based on Python trackpy v.0.4.2 library. First, particle tracking was performed; then, the tracks belonging to leukocytes were selected manually. The platelet thrombi location was determined using ilastik (http://ilastik.org) pixel classifier. Platelet thrombi area was calculated as the percentage of the screen covered by platelet thrombi. Tracking Code listing and program operation examples are available at (https://github.com/juliajessika/Leukocytes2023).

The first step is the segmentation of thrombi and leucocytes from the background to produce binary (black and white) images. To do this, we use a pixel classifier trained within ilastik. Various pixel-level features including smoothed intensity and edge indicators are measured and used to train a random forest classifier with two outcomes: signal and background. Training images should be selected to ensure the full variability within the dataset is captured. Having trained the pixel classifier within ilastik, it is run on the full dataset.

### Statistics

All experiments were performed at least in triplicate with whole blood from 3 different adult, 3 different pediatric donors, and 6 SDS patients. Statistical analysis was performed using Python 3.6; all statistical details are provided in the figure legends.

### Computational modeling and algorithms

The mathematical model which describes the propagation of a chemoattractant in a flow is a system of differential equations integrated using the finite element method implemented in the COMSOL Multiphysics software (COMSOL Multiphysics® v. 5.4. www.comsol.com. COMSOL AB, Stockholm, Sweden). Briefly, we solved the Navier–Stokes equation under the assumption of laminar flow and then calculated the distribution of chemoattractants by solving reaction–diffusion-advection equation. Equations and modeling details are given in the Additional file 1: S1, Table S1. References: [30, 31, 64, 65].

For further analysis, we developed a stochastic algorithm simulating neutrophil movement within the previously computed chemokine field based on an existing model by Szatmary et al. [4]. In this model, neutrophil movement is segmented into discrete timesteps. At each timestep, every neutrophil assesses chemokine concentrations at its current position within the gradient and then orients itself in alignment with the gradient. Within the model, it is posited that neutrophils move at a uniform velocity in their newly determined directions during each timestep. The decision is driven by the differential receptor occupancy (DFRO). This DFRO represents the variance in the proportion of receptors bound by ligands over the length of the migrating cell. The DFRO is defined by the following equation:

$$\mathrm{DFRO}\;=\;\frac{\ell_c}{K_d}\frac{dc}{dx}\frac1{{(c/\;K_d+1)}^2},$$

where *c* is the chemoattractant concentration, *K*_*d*_ is the dissociation constant for the chemoattractant-receptor interaction, and 𝓁_C_ is the length of the cell [4]. The chemoattractant gradient sampling was performed using Sobel matrices [66]. The image was rotated, so neutrophil movement direction on the previous step would align with *x*-axis. Sobel kernel size for *x*-axis was taken equal to average neutrophil length ${{\ell}}_{c}$ in pixels. Sobel kernel size for *y*-axis was taken equal to ${{\ell}}_{c}/2$, to indicate leucocyte ellipsoid shape. A random bias ${\theta }_{random}$ was introduced into neutrophil movement assuming that neutrophils’ orientations fall on a von Mises–Fisher distribution [67]. This is used to represent the observation that stronger gradient signals (i.e., higher DFRO) cause cell orientations to be more biased toward the gradient direction. The von Mises–Fisher distribution is given by $f({\theta }_{random};\kappa )=\frac{{\text{exp}}(\kappa {\text{cos}}({\theta }_{random}))}{2\pi {l}_{0}(\kappa )}$, where $\kappa =k \star DFRO$, where *k* is the sensitivity constant, and *l*_*0*_*()* is the modified Bessel function of zero order. Based on these quantities, we calculate the components of the model neutrophil velocity on the step *N* by a recurrent formulae:

$${v_x^N}_=\frac1{1+B_{mem}}v_{avg}^cos{\theta_{sum}^N}_+\frac{B_{mem}}{1+B_{mem}}v_{avg}^cos\theta_{sum}^{N-1},$$

$${v_y^N}_=\frac1{1+B_{mem}}v_{avg}^sin{\theta_{sum}^N}_+\frac{B_{mem}}{1+B_{mem}}v_{avg}^sin\theta_{sum}^{N-1},$$

where *θ*_*sum*_ = *θ*_*grad*_ + *θ*_*random*_, *θ*_*grad*_ is the direction of the gradient, ${B}_{mem}=L/(\triangle t)$ is neutrophil memory coefficient, ${v}_{avg}$ is the neutrophil average velocity, *L* is neutrophil persistence length [4], and * △t* is model time step. We chose ${v}_{avg}$ corresponding to the experimental average velocity of each neutrophil. Parameters such as ${{\ell}}_{c}$ and L were chosen for each leucocyte independently to fit the experimental trajectory as good as possible. Persistence length *L* was chosen to be in the same order of magnitude as in Vicker et al. [49] ($L=22 s$). Neutrophil size ${{\ell}}_{c}$ was chosen from the range of 10–20 μm [68]. The described algorithm was implemented in Python 3.6.

## Supplementary Information

Additional file 1: Figures S1-S12, Information S1, Table S1. Information S1: Equations and modeling details for the chemoattractant distribution model. Table S1: Parameters of the blood used for the mathematical model of chemokine distribution in flow chambers. Figure S1: Observation of NET formation in blood plasma smears or flow chambers. Representative images. Figure S2. Area, covered with thrombi, does not correlate with neutrophil velocities for SDS patients. Figure S3. Parameters of the flow chambers. A trajectory of neutrophil movement, flow direction, and the investigated region in the flow chamber are depicted. Figure S4. The distance from the nearest thrombus edge to the center of neutrophil compared to the distance from the thrombus border to a randomly placed, neutrophil-sized circle. Healthy donors, shear stress 100 s^−1^. Figure S5. The distance from the nearest thrombus edge to the center of neutrophil compared to the distance from the thrombus border to a randomly placed, neutrophil-sized circle. SDS patients, shear stress 100 s^−1^. Figure S6. Thrombus formation and neutrophil motility in blood samples from adult healthy donors (*n* = 6) for different wall shear rates. Figure S7. The distance from the nearest thrombus edge to the center of neutrophil compared to the distance from the thrombus border to a randomly placed, neutrophil-sized circle. Healthy donors, shear stress 200 s^−1^ or 300 s^−1^. Figure S8. Neutrophil motility parameters in stopped flow Figure S9. Thrombus formation and neutrophil motility in blood samples from adult healthy donors (*n* = 4) for flow chambers with different matrix proteins. Figure S10. Supplemental experiments for investigation of cellular and non-cellular blood components’ impact on neutrophil motility. Figure S11. Individual model runs. Figure S12a. Model chemokine distribution around thrombi in flow chamber in the same conditions as in Fig. 5. Figure S12b. Individual model runs for two values of the CA diffusion coefficient (D).

Additional file 2. Individual data values for Figs. 1, 2, 3, 4.

Additional file 3: Individual data values for Figures S1, S2, S3, S4, S5, S6, S7, S8, S9, S10.

## Data Availability

All data generated or analyzed during this study are included in this published article, its supplementary information files, and publicly available repositories. Python code for the calculation of the crawling granulocyte velocity checking neutrophil movement directions and neutrophil to thrombi distance and code generated for mathematical model of neutrophil chemotaxis is uploaded to GitHub repository (minimal: python 3.4; platform 318 independent; requirements—trackpy and anaconda packages for python 3.4 and above): https://github.com/juliajessika/Leukocytes2023/. Raw microscopy data for NET-osis in blood smears could be found at figshare [69]. Raw microscopy data for NET-osis in flow chambers could be found at figshare [70]. Raw microscopy data for granulocyte movements could be found at OSF [71]. Raw values are given in Additional files 2 and 3.

## Abbreviations

SDS: Shwachman-Diamond syndrome
vWF: Von Willebrand factor
IL-8: Interleukin-8
LTB4: Leukotriene B4
CXCL1: Chemokine (C-X-C motif) ligand 1
ADP: Adenosine diphosphate
fMLP: N-Formylmethionyl-leucyl-phenylalanine
PFA: Paraformaldehyde
PBS: Phosphate-buffered saline
NET: Neutrophil extracellular trap
MDS: Myelodysplastic syndromes
AML: Acute myeloid leukemia
rhG-CSF: Recombinant human granulocyte colony-stimulating factor
ANC: Absolute neutrophil count
PPP: Platelet poor plasma
LRP: Leukocyte-rich plasma
BSA: Bovine serum albumin
MPO: Myeloperoxidase
NE: Neutrophil elastase
DFRO: Differential receptor occupancy
RBC: Red blood cell
CA: Chemoattractant
