# Supplementary material for: Dissecting thrombus-directed chemotaxis and random movement in neutrophil near-thrombus motion in flow chambers
Source: BMC Biol. 2024 May 20;22:115. doi: 10.1186/s12915-024-01912-2 (PMC11552338; doi:10.1186/s12915-024-01912-2)
Supplement: Supplementary file 1 — Additional file 1: Figures S1-S12, Information S1, Table S1. Information S1: Equations and modeling details for the chemoattractant distribution model. Table S1: Parameters of the blood used for the mathematical model of chemokine distribution in flow chambers. Figure S1: Observation of NET formation in blood plasma smears or flow chambers. Representative images. Figure S2. Area, covered with thrombi, does not correlate with neutrophil velocities for SDS patients. Figure S3. Parameters of the flow chambers. A trajectory of neutrophil movement, flow direction, and the investigated region in the flow chamber are depicted. Figure S4. The distance from the nearest thrombus edge to the center of neutrophil compared to the distance from the thrombus border to a randomly placed, neutrophil-sized circle. Healthy donors, shear stress 100 s−1. Figure S5. The distance from the nearest thrombus edge to the center of neutrophil compared to the distance from the thrombus border to a randomly placed, neutrophil-sized circle. SDS patients, shear stress 100 s−1. Figure S6. Thrombus formation and neutrophil motility in blood samples from adult healthy donors (n = 6) for different wall shear rates. Figure S7. The distance from the nearest thrombus edge to the center of neutrophil compared to the distance from the thrombus border to a randomly placed, neutrophil-sized circle. Healthy donors, shear stress 200 s−1 or 300 s−1. Figure S8. Neutrophil motility parameters in stopped flow Figure S9. Thrombus formation and neutrophil motility in blood samples from adult healthy donors (n = 4) for flow chambers with different matrix proteins. Figure S10. Supplemental experiments for investigation of cellular and non-cellular blood components’ impact on neutrophil motility. Figure S11. Individual model runs. Figure S12a. Model chemokine distribution around thrombi in flow chamber in the same conditions as in Fig. 5. Figure S12b. Individual model runs for two values of the CA diffusion coefficient [file 12915_2024_1912_MOESM1_ESM.pdf]

Supporting Information

Dissecting Thrombus-Directed Chemotaxis and Random Movement in Neutrophil Near-Thrombus Motion in Flow Chambers

Julia-Jessica D. Korobkin[a], Ekaterina A. Deordieva[b], Ivan P. Tesakov[b,c], Ekaterina A. Adamanskaya[a,b], Anna E. Boldova [a,b], Antonina A. Boldyreva[a,d], Sofia V. Galkina[a,b], Daria P. Lazutova[a], Alexey A. Martyanov[a], Vitaly A. Pustovalov[e], Galina A. Novichkova[b], Anna Shcherbina[b], Mikhail A. Panteleev[a,b,f], and Anastasia N. Sveshnikova[a,b,f,*]

[a] Center for Theoretical Problems of Physico-Chemical Pharmacology, Russian Academy of Sciences, Moscow, Russia

[b] Dmitry Rogachev National Medical Research Center of Pediatric Hematology, Oncology and Immunology, Moscow, Russia

[c] Department of Oncology, Hematology, Immunology, and Rheumatology, University Hospital Tübingen, Tübingen, Germany

[d] Sechenov First Moscow State Medical University, Moscow, Russia

[e] Federal Institute of Technology Zurich, Zürich, Switzerland

[f] Lomonosov Moscow State University, Moscow, Russia

Short Title: Chemotaxis in neutrophil near-thrombus movement

Corresponding Author:

Anastasia Sveshnikova

E-mail address: a.sveshnikova@physics.msu.ru

Keywords: chemotaxis, hemostasis, neutrophils , platelets, thromboinflammation

S1. Equations and modeling details for the chemoattractant distribution model

For modeling the chemoattractant distribution around the growing thrombi, first, the respective Navier-Stokes equation in the assumption of laminar flow was solved:

$$\rho\left[\frac{\partial}{\partial t}+\mathbf{v}\,\mathrm{grad}\right]\mathbf{v}=\mathbf{f}-\mathrm{grad}\,p+\ \eta\Delta\mathbf{v}.\ (1)$$

Scheme of the model is shown in Figure S1b. For the Navier-Stokes equation, we applied the following boundary conditions:

1. *p*=0 at the liquid outlet,
2. *v*=0.05 m/s at the liquid inlet (both are indicated with red arrows in Fig. S1b),
3. Dirichlet conditions are imposed on the surfaces of the flow chamber (indicated with blue crosses in Fig. S1b)
4. Periodic boundary conditions are imposed on surfaces denoted by a yellow cross in Figure S1b.

We simulated the liquid flow within a volume measuring $h*h*d$, where h varied from 250 to 350 µm, corresponding to the part of the field of view in the microscopy video around the neutrophil, and d = 100 µm, corresponding to the thickness of the flow chamber. Individual platelets were denoted as spheres with a diameter of 1 µm, and Dirichlet boundary conditions were imposed on their surfaces. All the necessary physical parameters for the liquid and chemokines, such as blood density, dynamic viscosity, diffusion coefficient, inlet velocity, and initial concentration, were either sourced from the literature or calculated from experimental data (Table S1).

Table S1. Parameters of the blood used for the mathematical model of chemokine distribution in flow chambers

Parameter	Value	Source
blood density	1060 kg/m^3	Ref 64
dynamic viscosity	5x10^{-3} Pa/s	Ref 65
diffusion coefficient	1.3x10^{-10} m^2/s	Ref 30
inlet velocity	0.05 m/s	
Chemoattractant flux	2900 molecules per duration of the experiment (30 min) per platelet	Ref 31

Supporting Figures

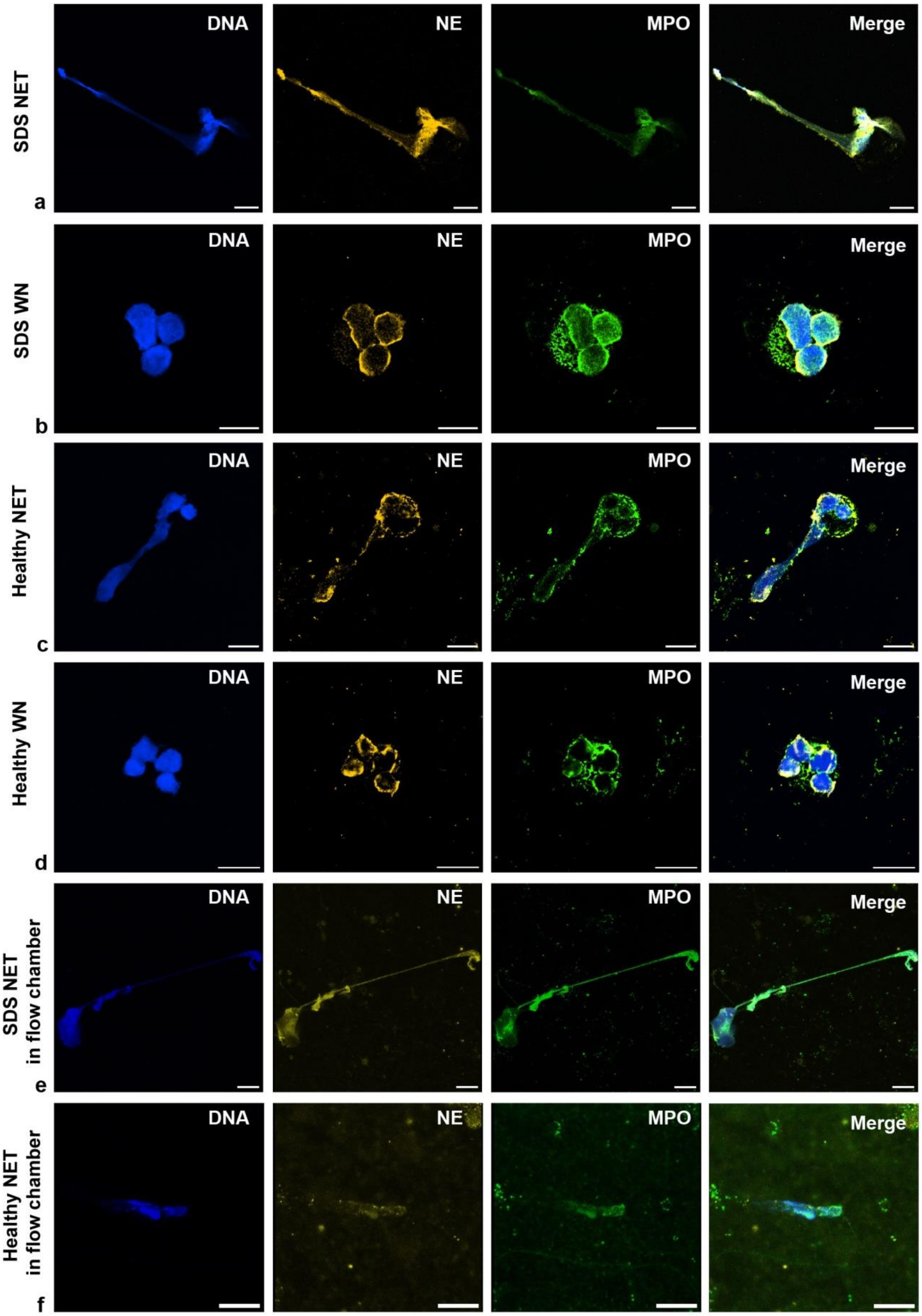

Figure S1. Observation of NET formation in blood plasma smears (a-d) or flow chambers (e,f). Representative images. Scale bar 10 µm. DNA – staining with Hoechst33342, NE – staining with antibodies against human neutrophil elastase, MPO – staining with antibody against human active myeloperoxidase. SDS - Shwachman-Diamond syndrome, WN – whole neutrophil.

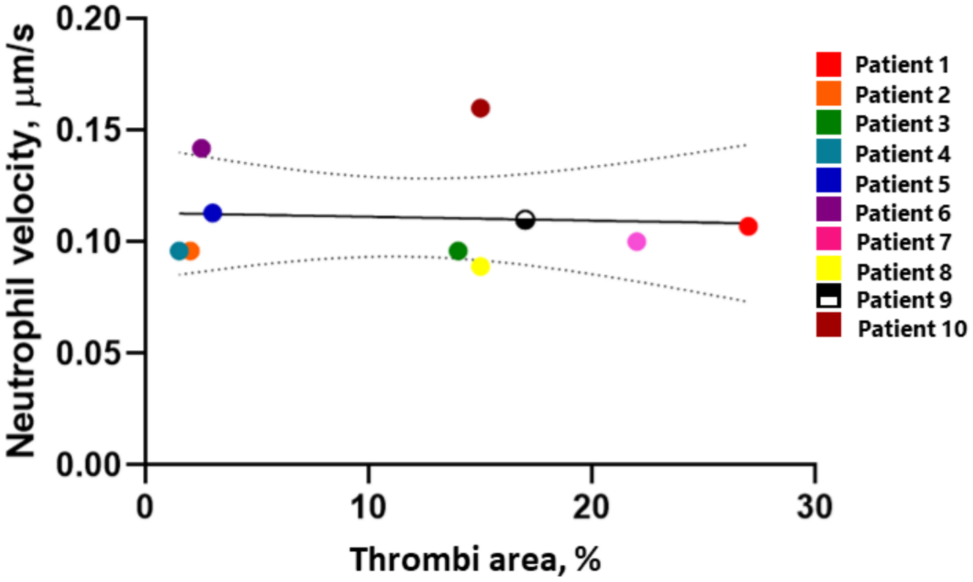

Figure S2. Area, covered with thrombi, does not correlate with neutrophil velocities for SDS patients (n =10). The Spearman correlation coefficient was computed, r = 0.09, p>0.99. Raw data values are given in Additional file 3:FigS2.

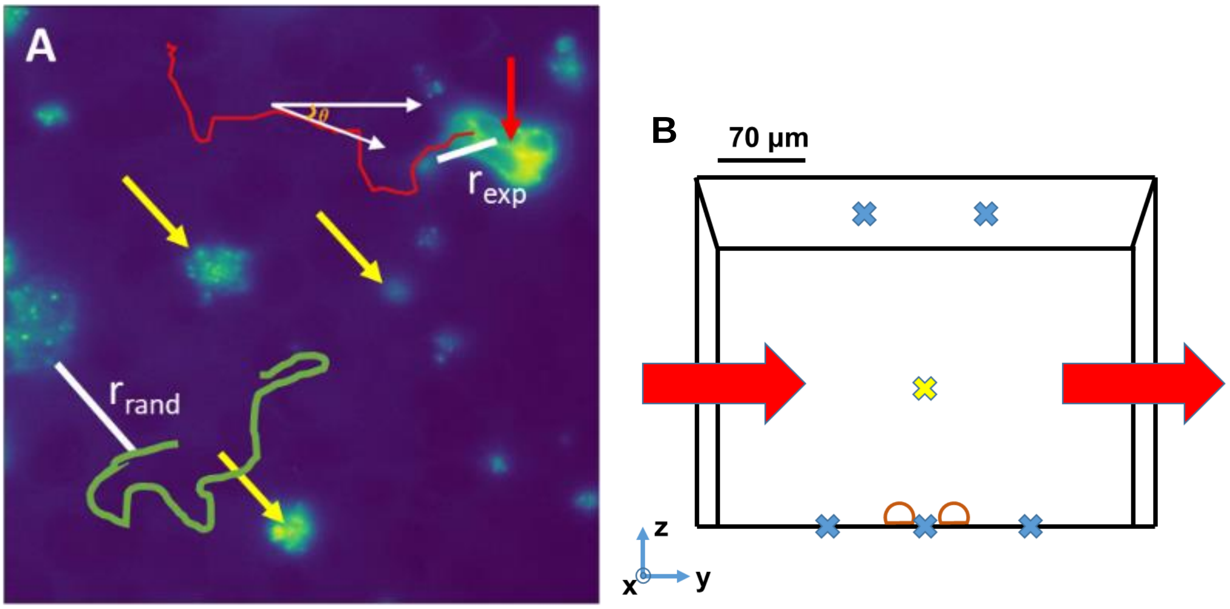

Figure S3. Parameters of the flow chambers. A. A trajectory of neutrophil movement is depicted with a red line, with the white arrow representing the flow direction. Random trajectory is depicted with a green line. Red arrow indicates a neutrophil. Yellow arrows indicate thrombi locations. The distances between neutrophil and thrombus, r_{rand} and r_{exp}, are shown as white bars. (B). The investigated region in the flow chamber. Flow direction is shown with the red arrows. Surfaces with Dirichlet boundary conditions are marked with blue crosses. Yellow crosses denote periodic boundary conditions. Red arrows indicate flow directions and inlet and outlet locations. Z-scale is not conserved.

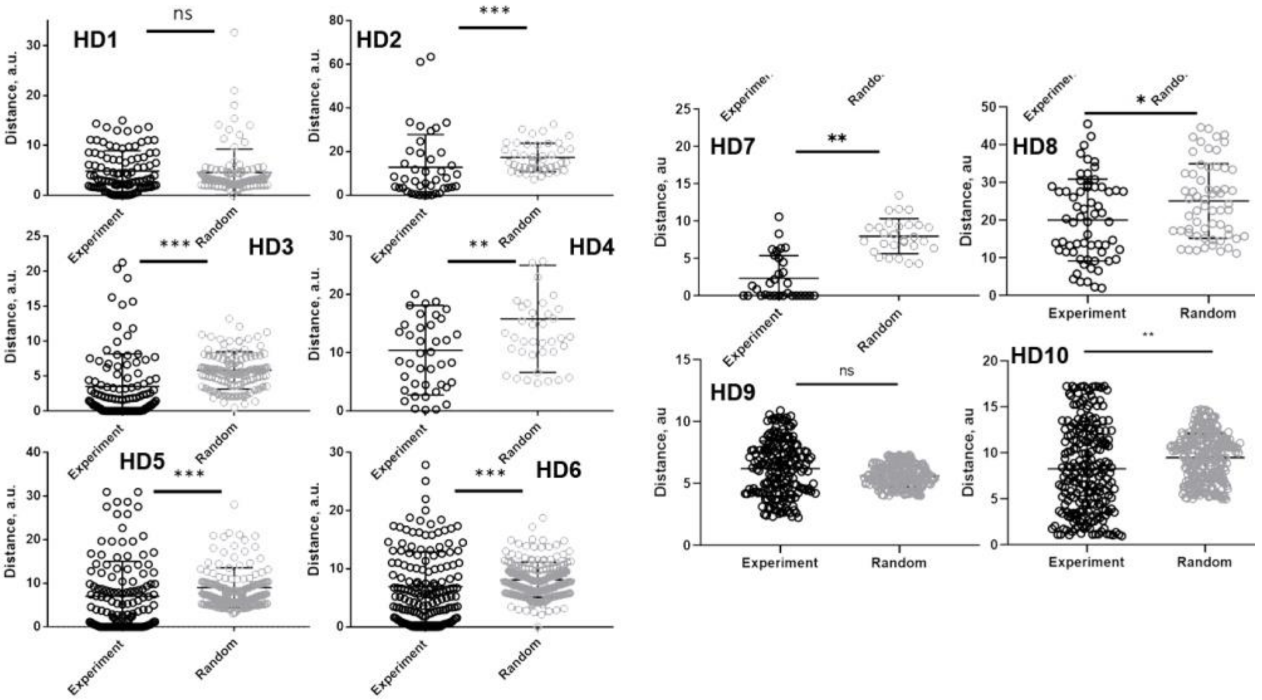

Figure S4. The distance from the nearest thrombus edge to the centre of neutrophil compared to the distance from the thrombus border to a randomly placed, neutrophil-sized circle. Healthy donors, shear stress 100 s^{-1}. * corresponds to $p < 0.05$; ** corresponds to $p < 0.01$; *** $p < 0.001$. Statistical significance was determined using the Mann-Whitney test. Raw data values are given in Additional file 3:FigS4.

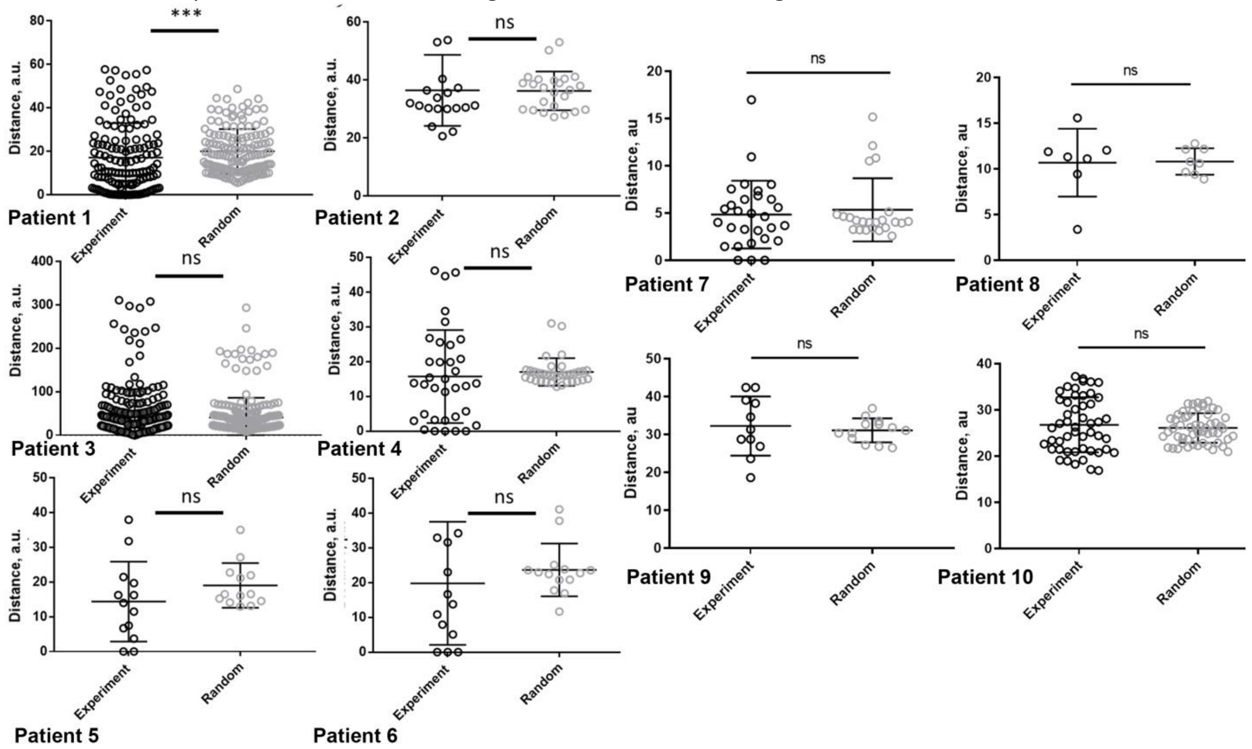

Figure S5. The distance from the nearest thrombus edge to the centre of neutrophil compared to the distance from the thrombus border to a randomly placed, neutrophil-sized circle. SDS patients, shear stress 100 s^{-1}. * - corresponds to $p<0.05$; ** - corresponds to $p<0.01$; *** - $p<0.001$. Statistical significance was calculated using Mann-Whitney test. Raw data values are given in Additional file 3:FigS5.

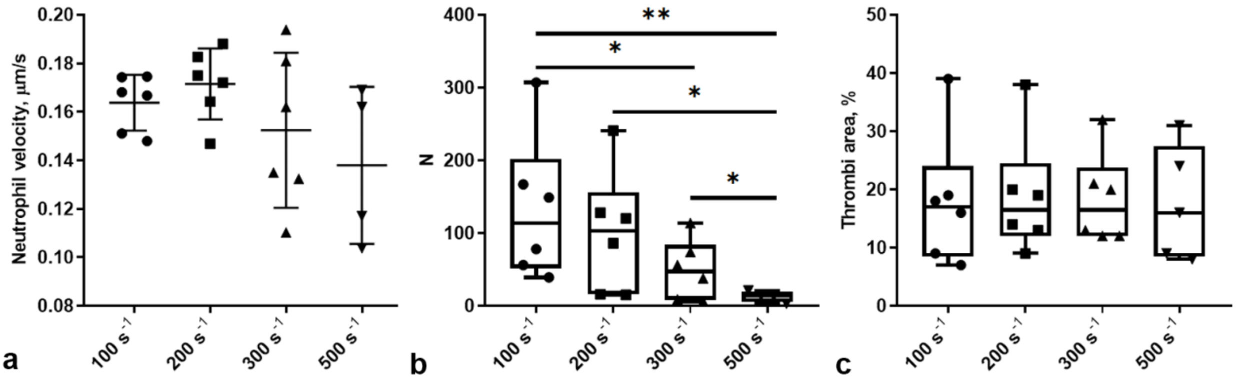

Figure S6. Thrombus formation and neutrophil motility in blood samples from adult healthy donors (*n* = 6) for different wall shear rates. (a) Average velocities of neutrophil movement were not influenced by flow. (b) Number of adhering neutrophils per experiment significantly reduced with increased flow rates. (c) Thrombus area was not affected by flow. Raw data values are given in Additional file 3:FigS6.* - corresponds to $p<0.05$; ** - corresponds to $p<0.01$; *** - $p<0.001$. Statistical significance was calculated using Mann-Whitney test.

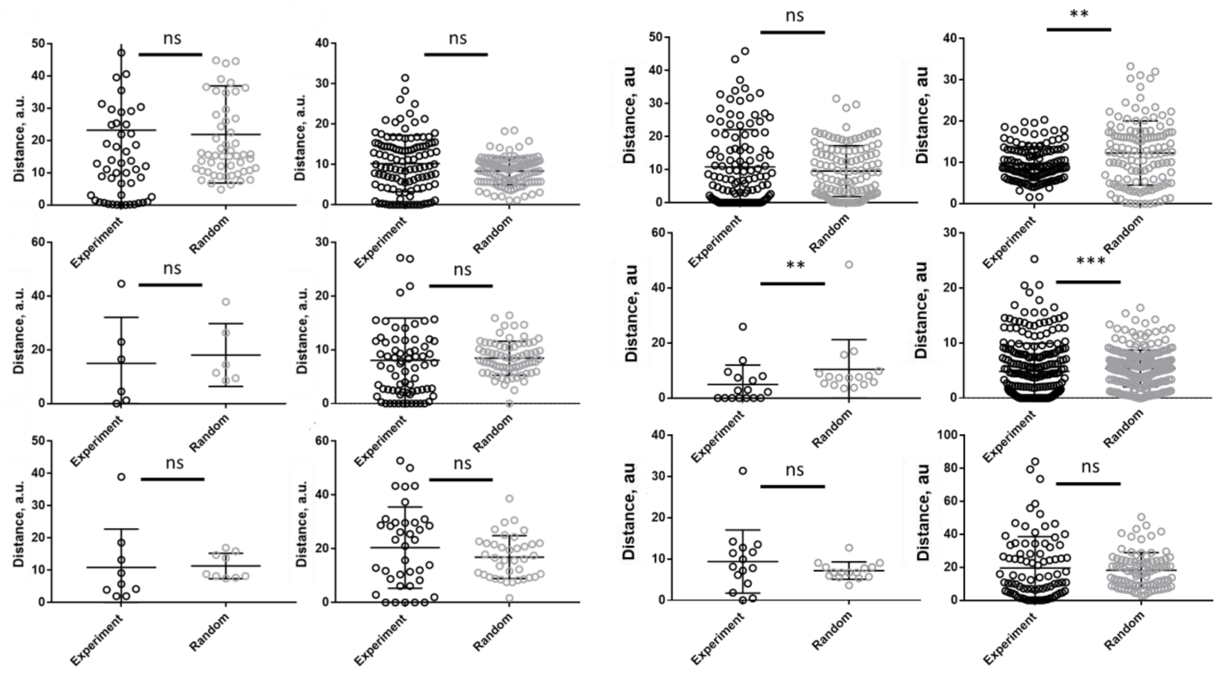

a *b*

Figure S7. The distance from the nearest thrombus edge to the centre of neutrophil compared to the distance from the thrombus border to a randomly placed, neutrophil-sized circle. Healthy donors, shear stress 200 s^{-1}(a) or 300 s^{-1}(b). * corresponds to $p < 0.05$; ** corresponds to $p < 0.01$; *** $p < 0.001$. Statistical significance was determined using the Mann-Whitney test. Raw data values are given in Additional file 3:FigS7.

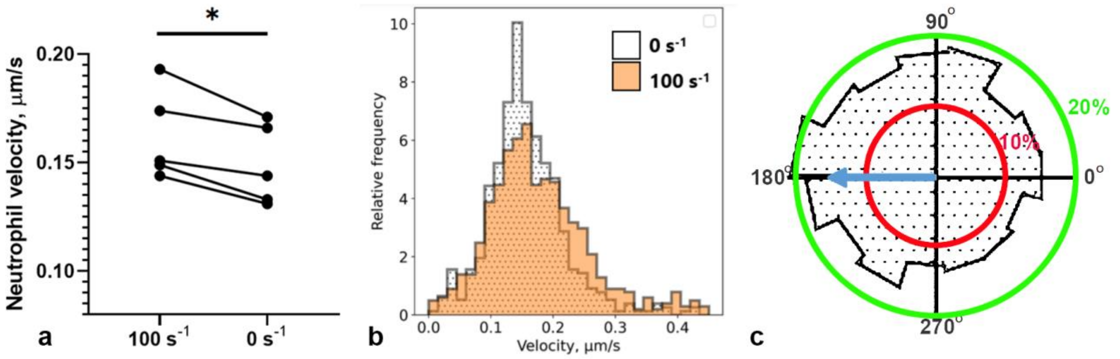

Figure S8. Neutrophil motility parameters in stopped flow. (a) Average velocities of neutrophil movement diminished upon turning off the flow. n = 5. * - corresponds to p<0.05. Paired t-test was used to calculate statistical significance. Raw data values are given in Additional file 3:FigS8. (b) Neutrophil velocities distribution for shear stress of 100 s^{-1} and without flow. (c) Histogram of neutrophil movement direction (θ) in samples from healthy donors on collagen coating for stopped blood flow. Blue arrow indicates residual flow direction. In healthy donors (n = 5, 60 neutrophil trajectories randomly picked from each donor, each trajectory resampled to be at least 250 seconds long), neutrophils predominantly move along the flow

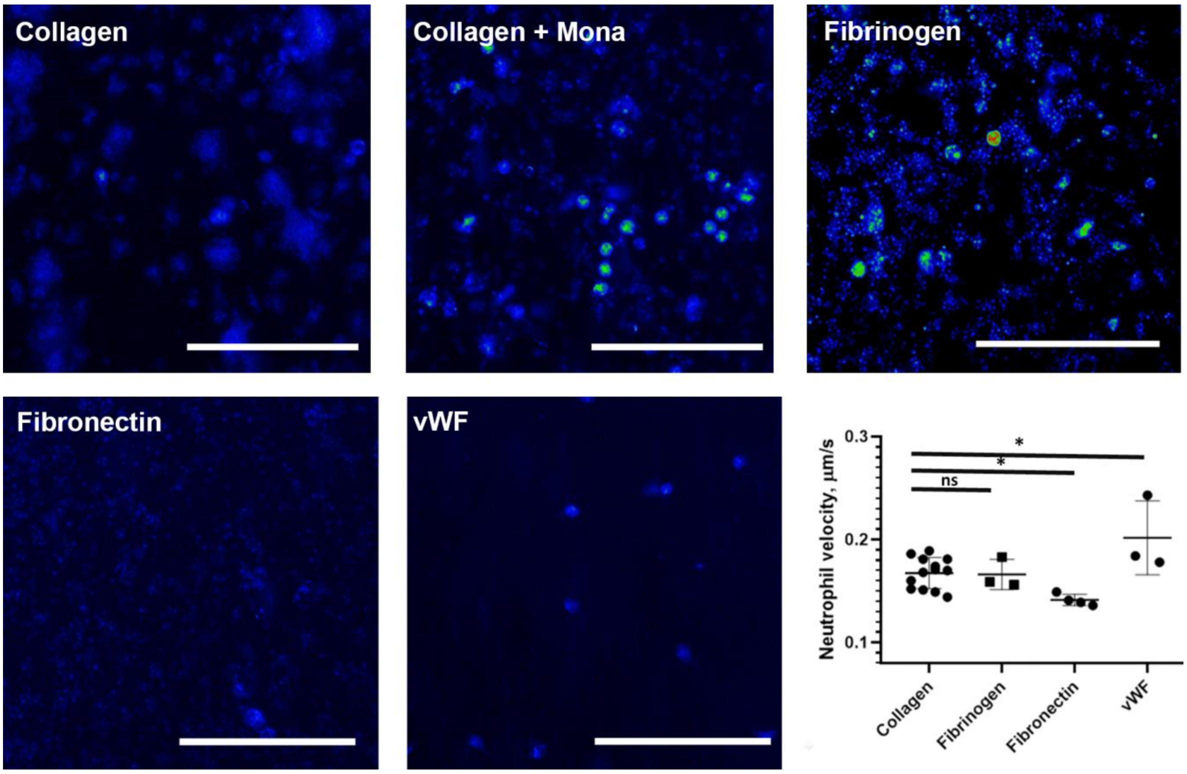

Figure S9. Thrombus formation and neutrophil motility in blood samples from adult healthy donors (*n* = 4) for flow chambers with different matrix proteins. Rainbow colouring indicates the intensity of DiOC6 staining, representative images. * - corresponds to p<0.05; Statistical significance was calculated using Mann-Whitney test. Raw data values are given in Additional file 3:FigS9.

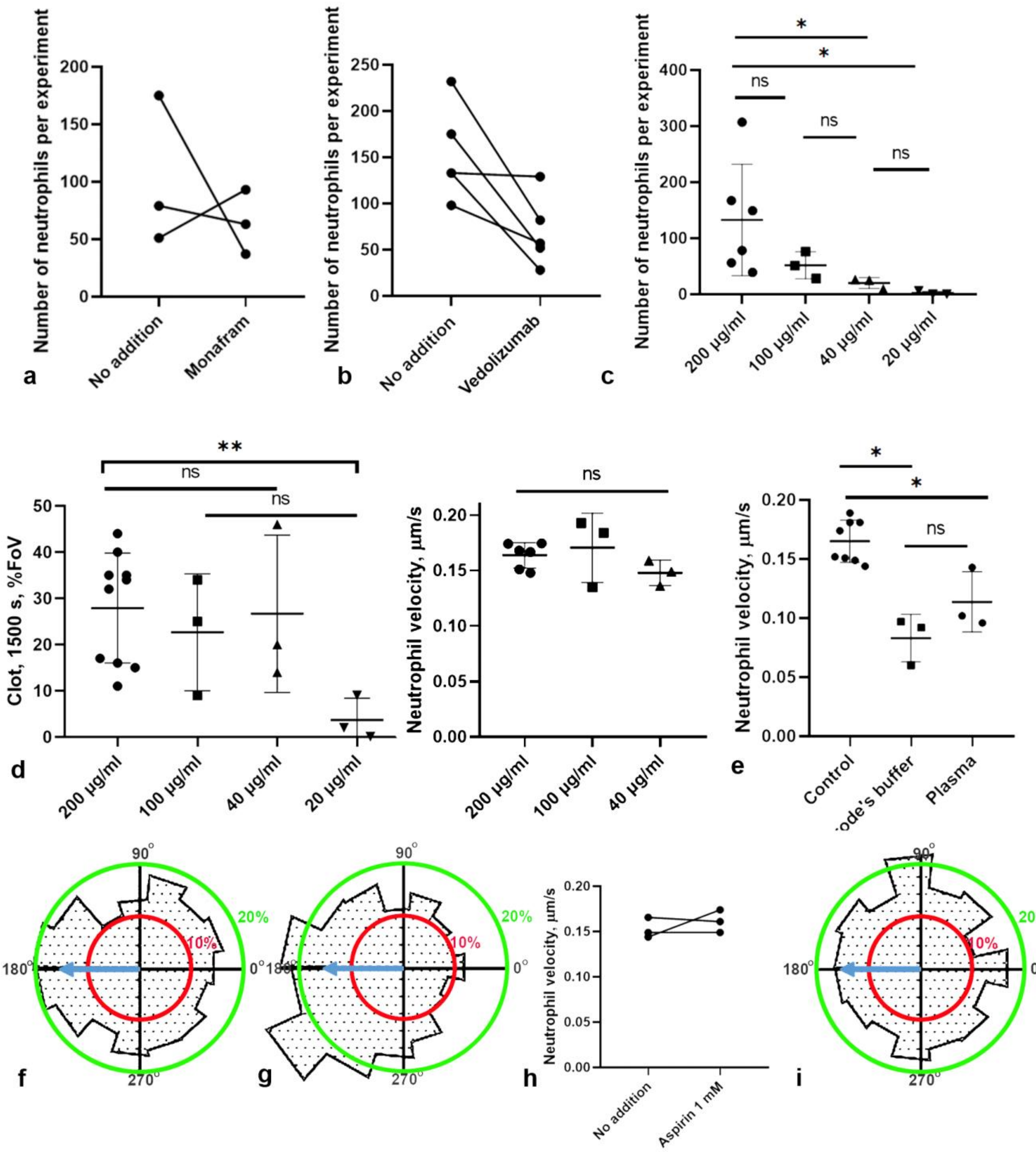

Figure S10. Supplemental experiments for investigation of cellular and non-cellular blood components' impact on neutrophil motility. (a-c) Numbers of neutrophils after 1500 s of healthy donors blood perfusion with shear rate 200 s^{-1} through flow chamber with collagen-coated coverslip. (a) Blood sample was pre-incubated with Monafram (n = 3). (b) Blood sample was pre-incubated with Vedolizumab (n = 5). (c) Different collagen concentrations (n = 6, 3). (d) Thrombi area and neutrophil velocities for the same experiments as in (c). (e-g) Same conditions, after 10 minutes of blood perfusion either Tyrodes buffer with calcium (e, f) or platelet poor plasma (e, g) were perfused through the system for 15 minutes. (e) Average velocities of neutrophil movement (n = 8, 3). (f,g) Histogram of neutrophil movement direction (θ). Blue arrow indicates flow direction. * corresponds to $p < 0.05$; ** corresponds to $p < 0.01$; *** $p < 0.001$. Statistical significance was determined using the Mann-Whitney test. (h,i) Same conditions as in (a), blood samples pre-incubated with 1mM of Aspirin for 30 min. (h) Average velocities of neutrophil movement (n = 3). Histogram of neutrophil movement direction (θ). Blue arrow indicates flow direction. Raw data values are given in Additional file 3:FigS10.

Figure S11. Individual model runs

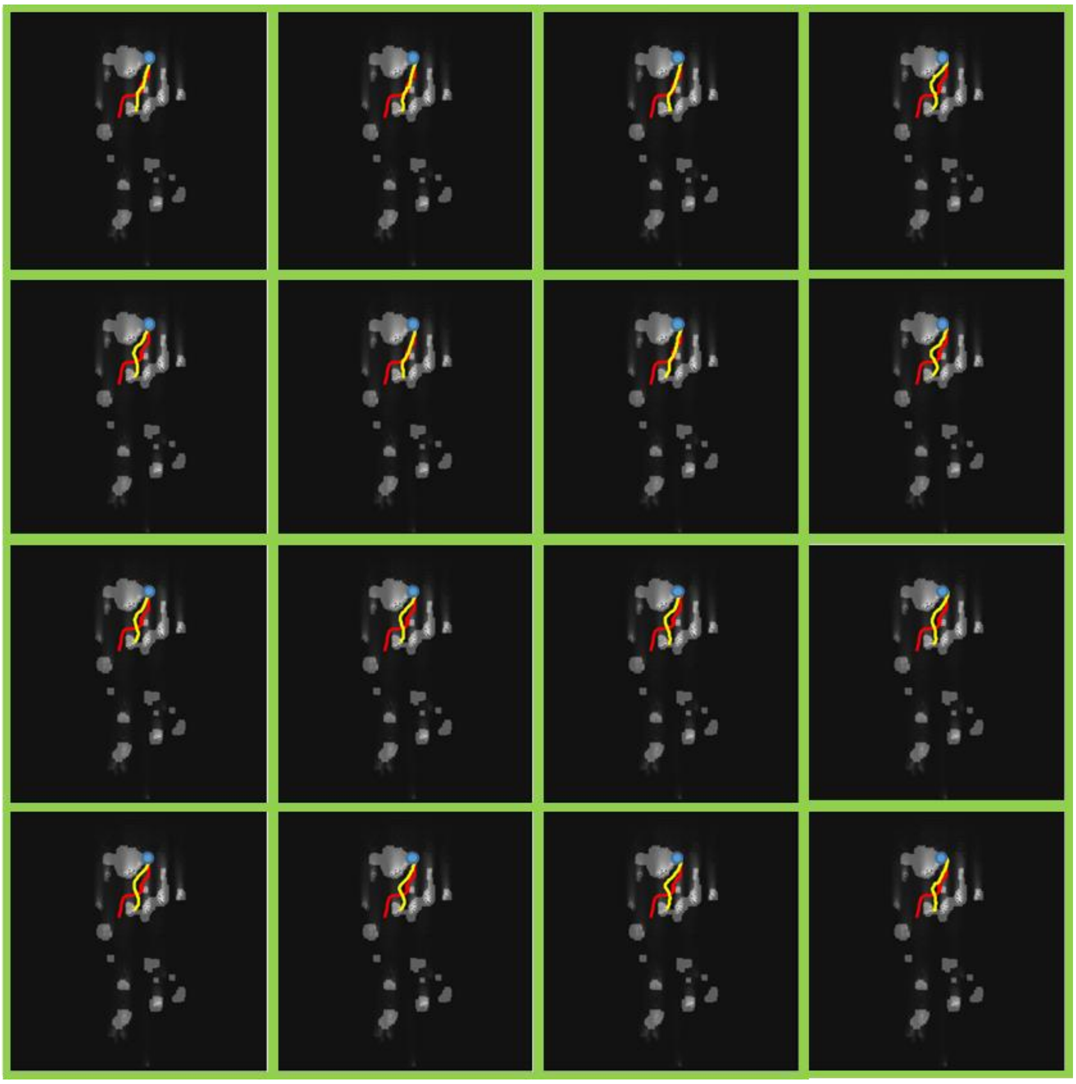

Healthy adult donor 1, trajectory 1. 16/16 model runs describe the experimental trajectory.

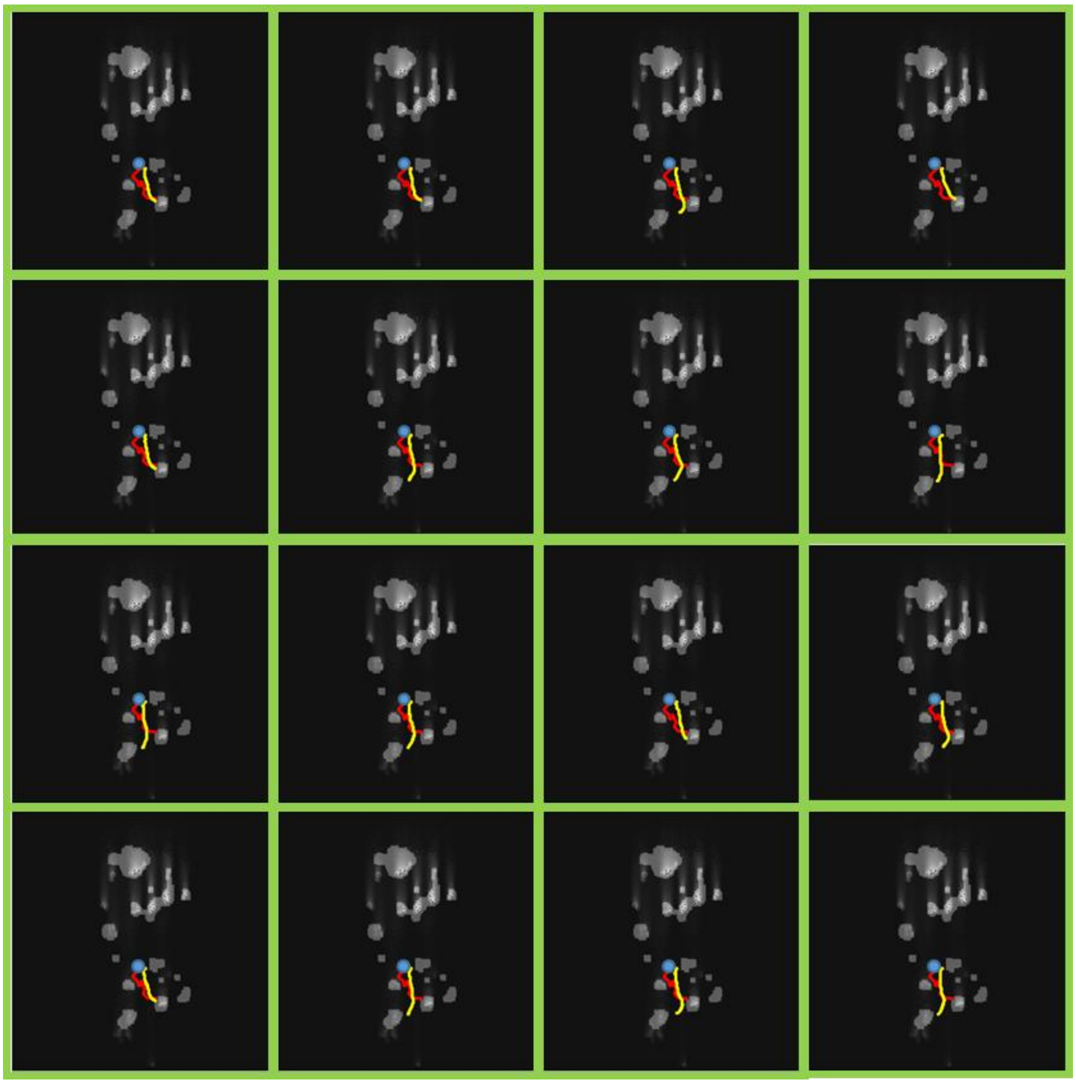

Healthy adult donor 1, trajectory 2. 16/16 model runs describe the experimental trajectory.

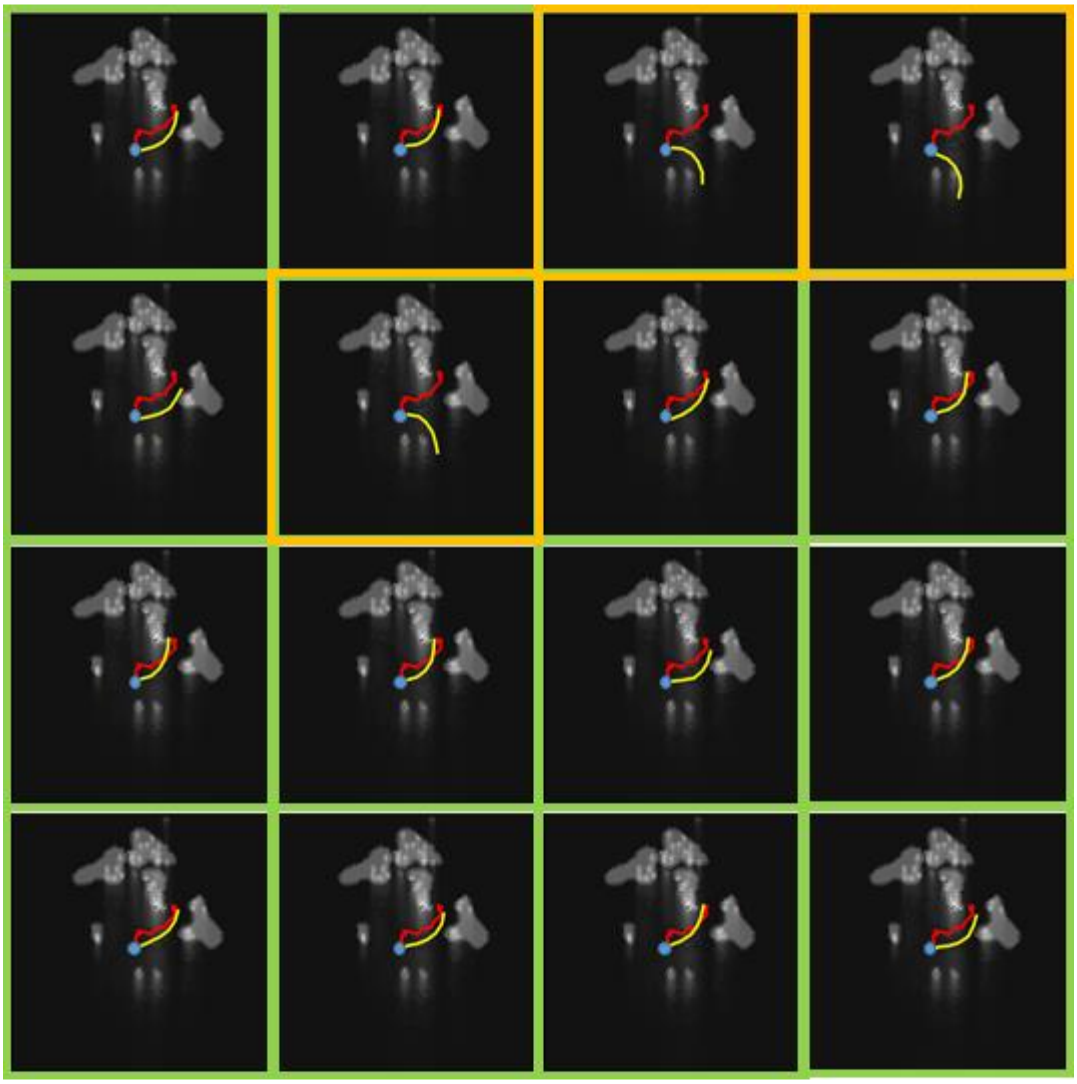

Healthy adult donor 1, trajectory 3. 13/16 model runs describe the experimental trajectory.

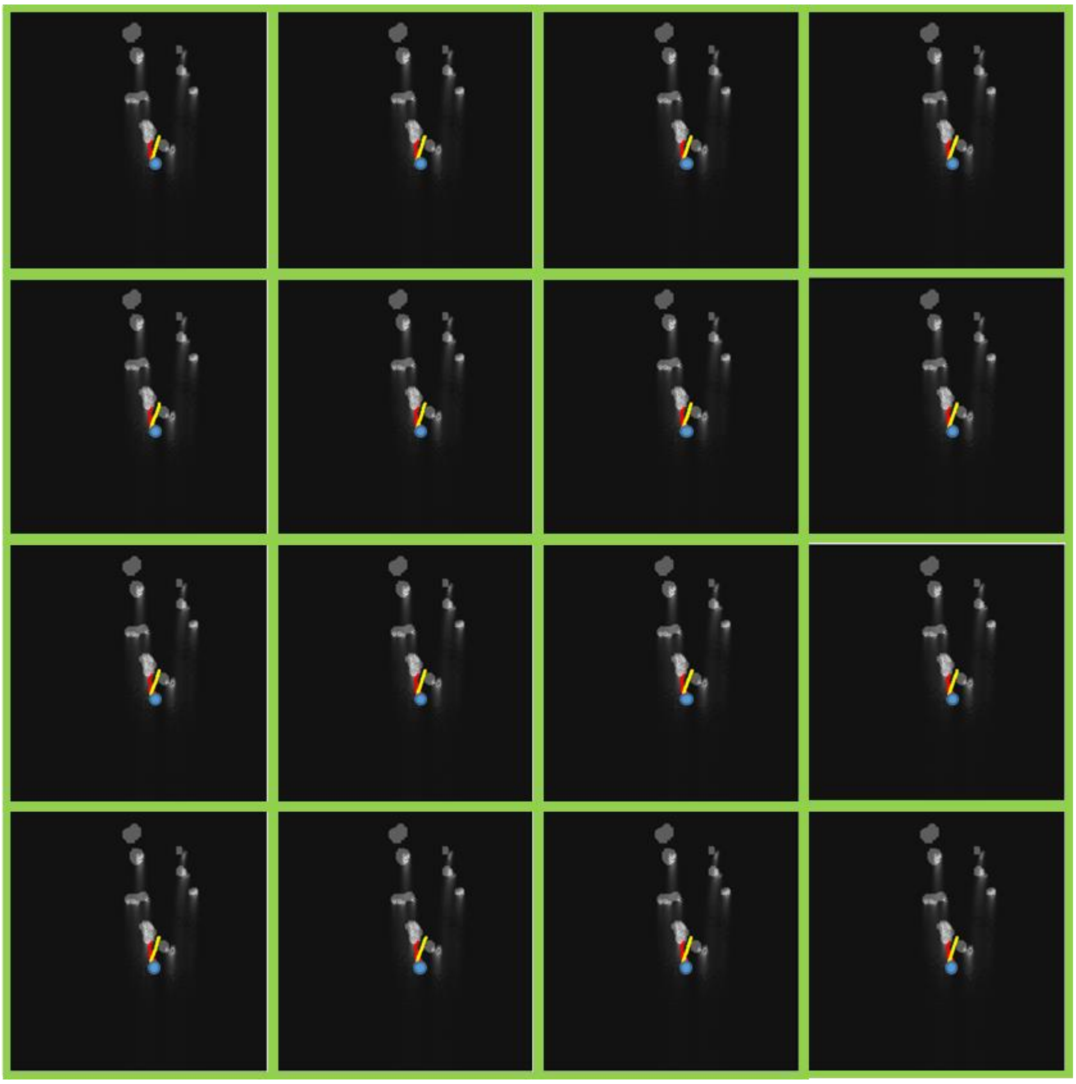

Healthy pediatric donor 1, trajectory 1. 16/16 model runs describe the experimental trajectory.

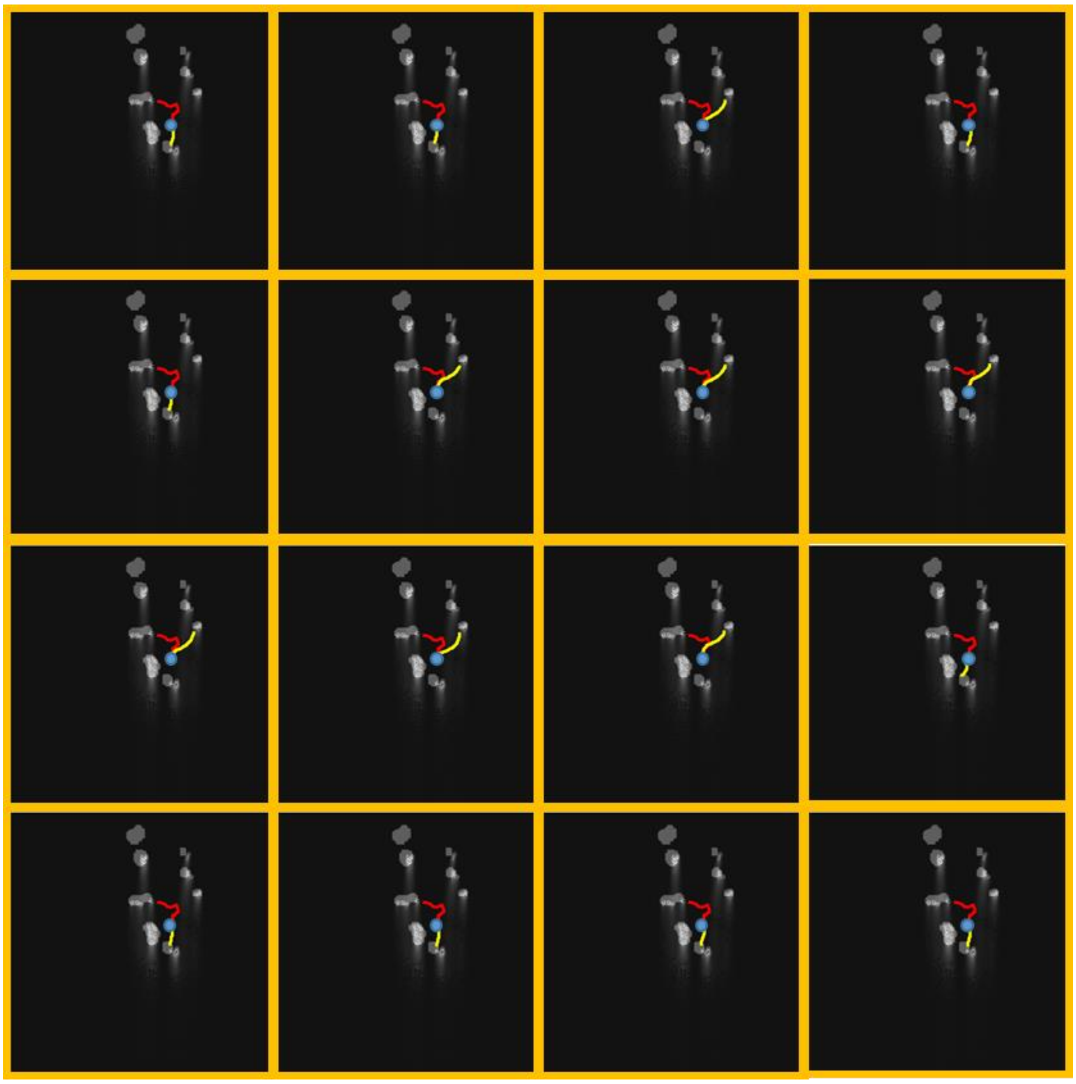

Healthy pediatric donor 1, trajectory 2. 0/16 model runs describe the experimental trajectory.

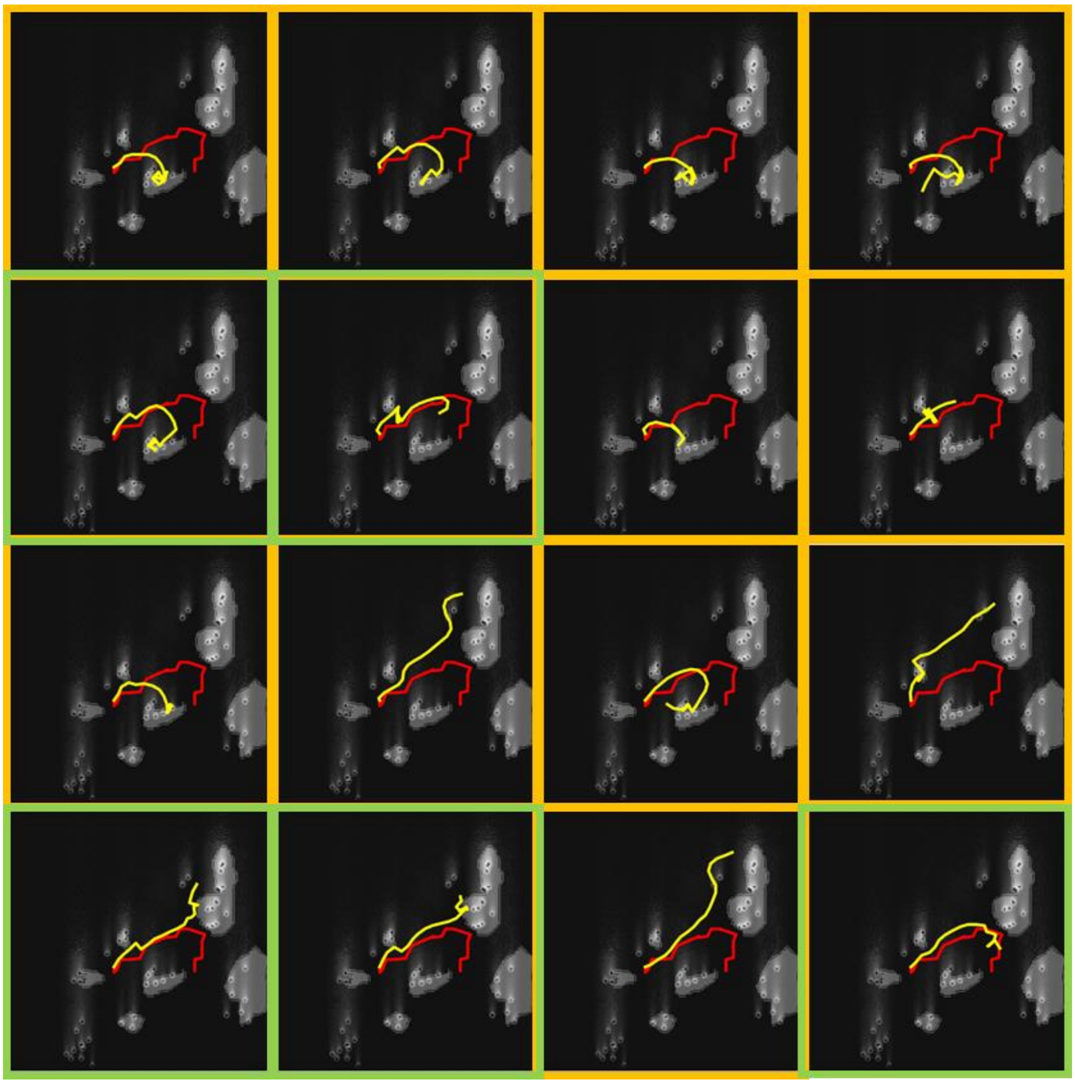

Healthy pediatric donor 1, trajectory 3. 6/16 model runs describe the experimental trajectory.

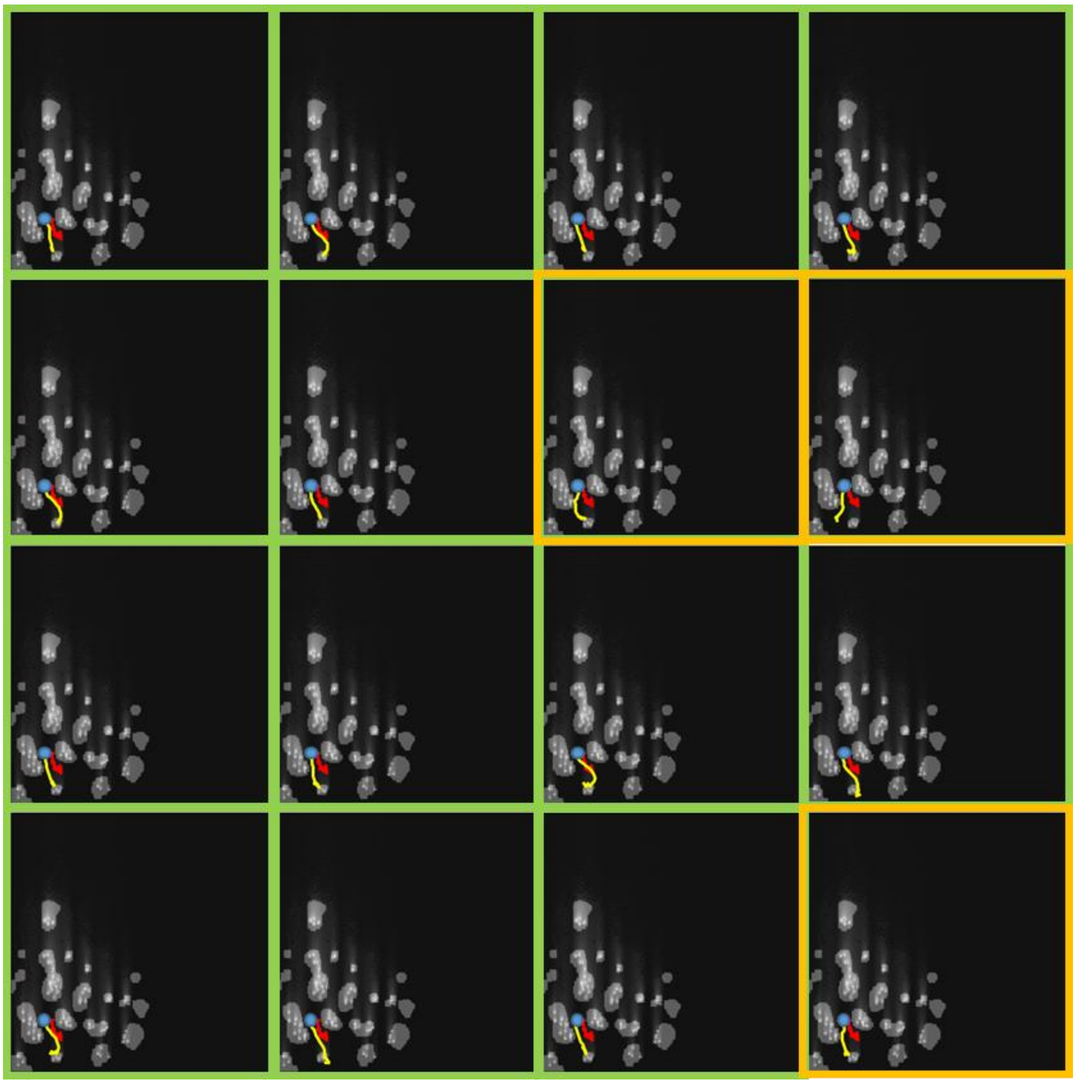

Healthy adult donor 2, trajectory 1. 16/16 model runs describe the experimental trajectory.

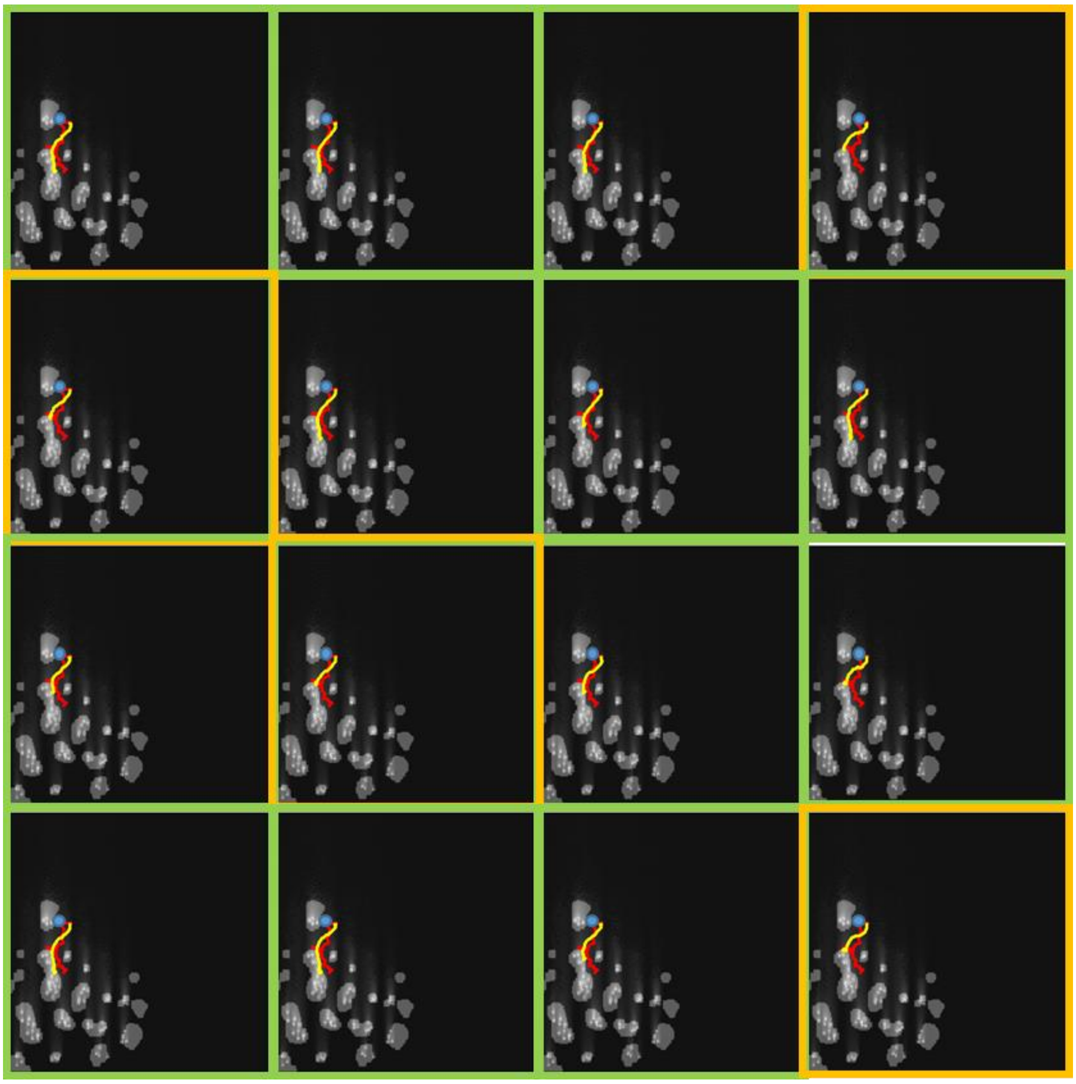

Healthy adult donor 2, trajectory 2. 12/16 model runs describe the experimental trajectory.

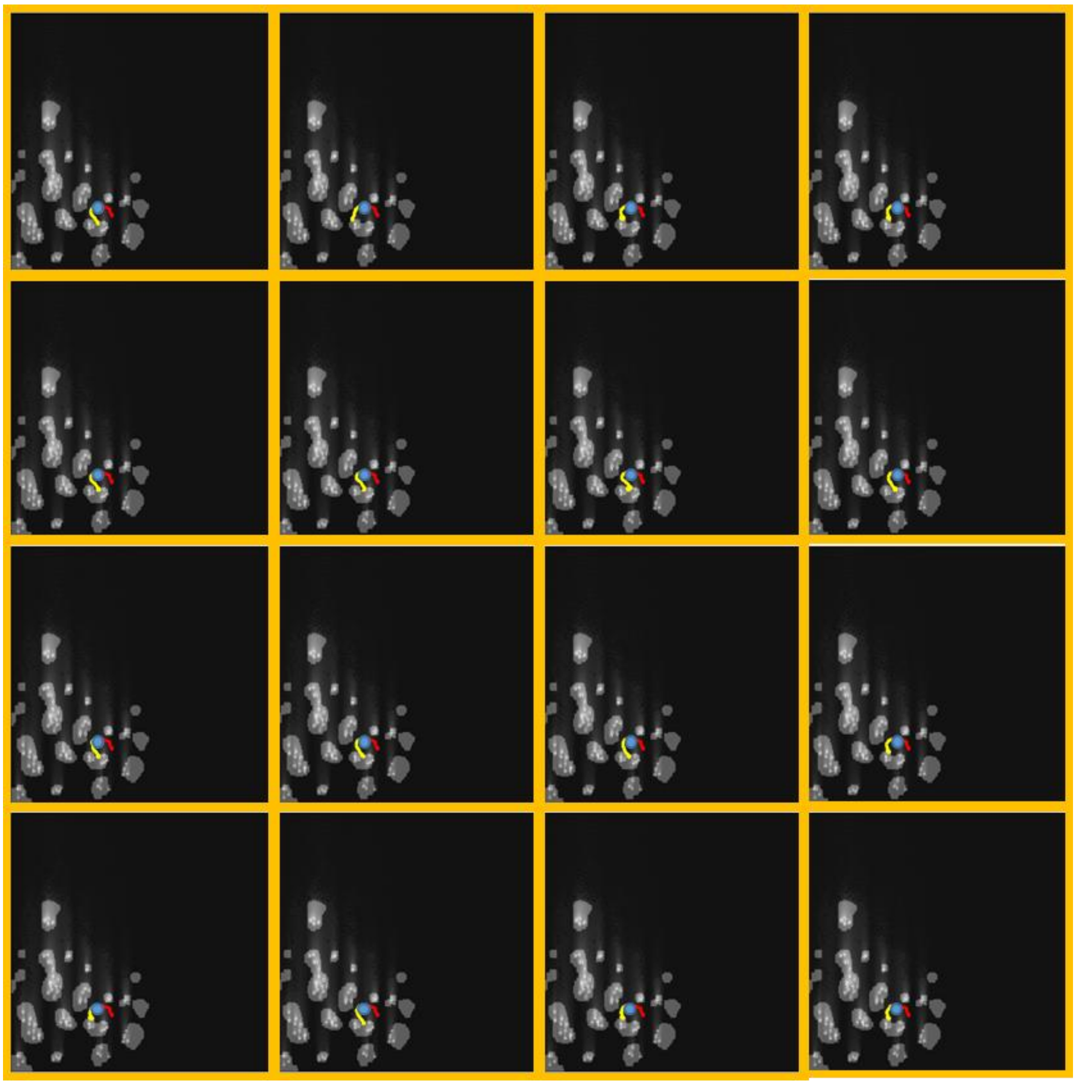

Healthy adult donor 2, trajectory 3. 0/16 model runs describe the experimental trajectory.

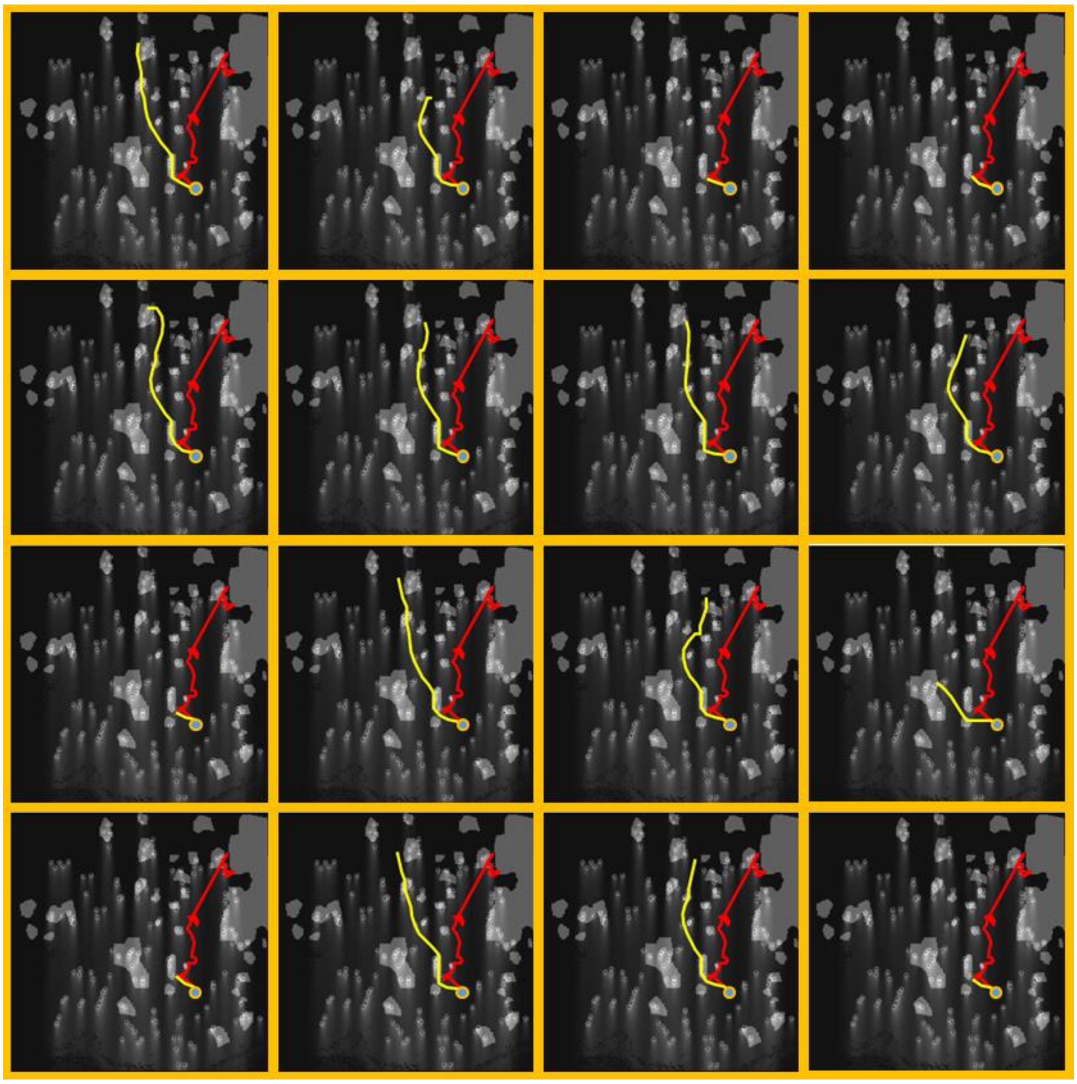

Healthy adult donor 3, trajectory 1. 0/16 model runs describe the experimental trajectory.

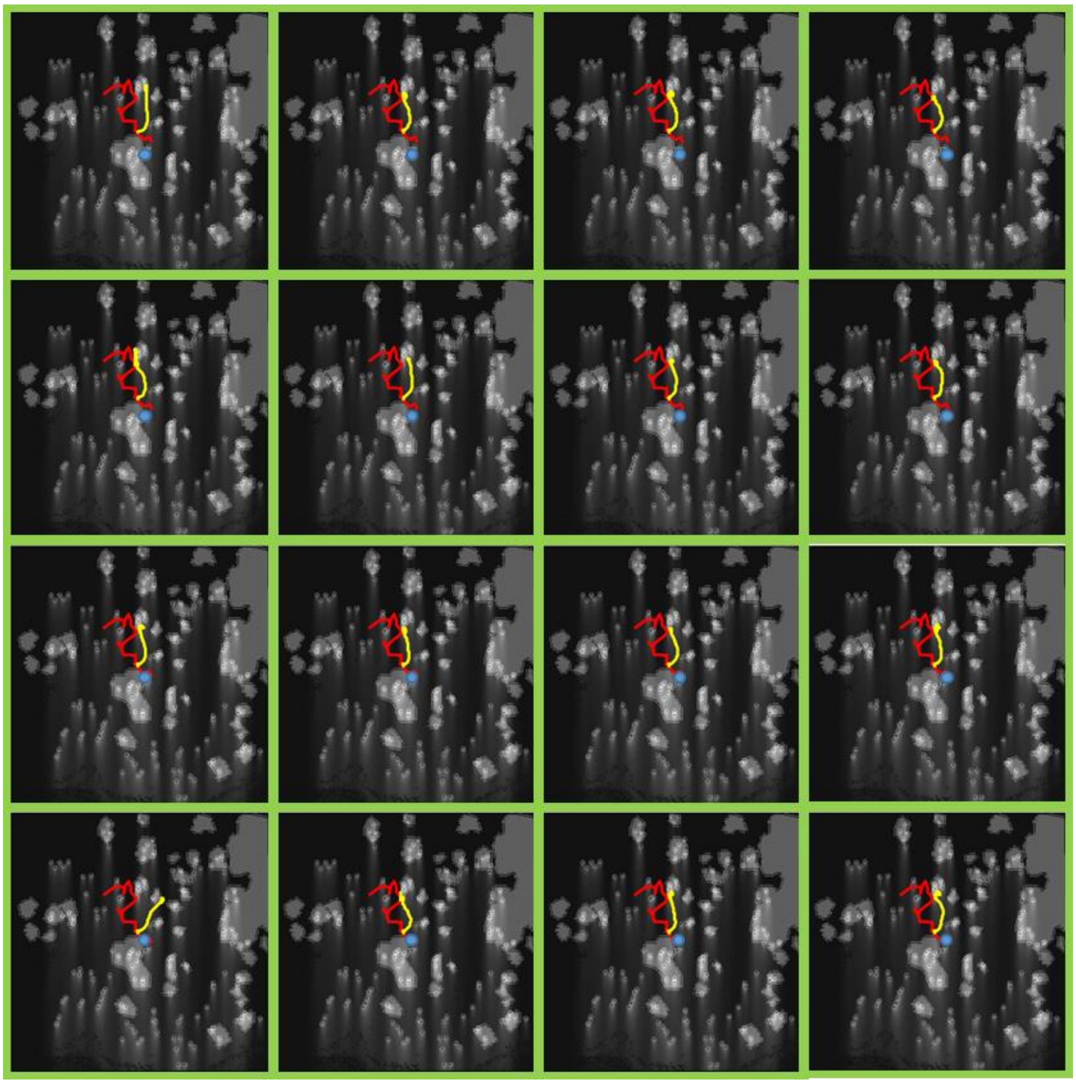

Healthy adult donor 3, trajectory 2. 16/16 model runs describe the experimental trajectory.

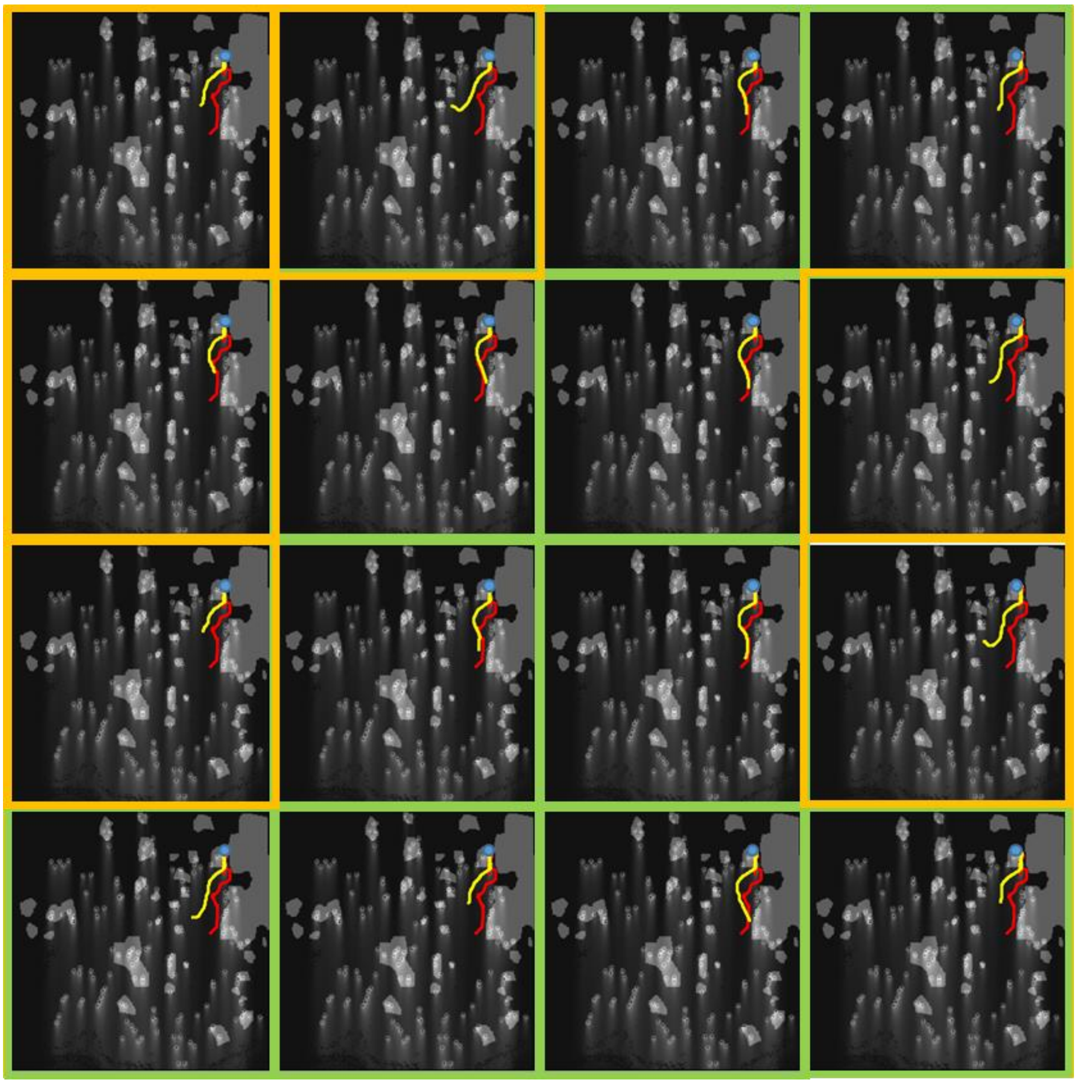

Healthy adult donor 3, trajectory 3. 10/16 model runs describe the experimental trajectory.

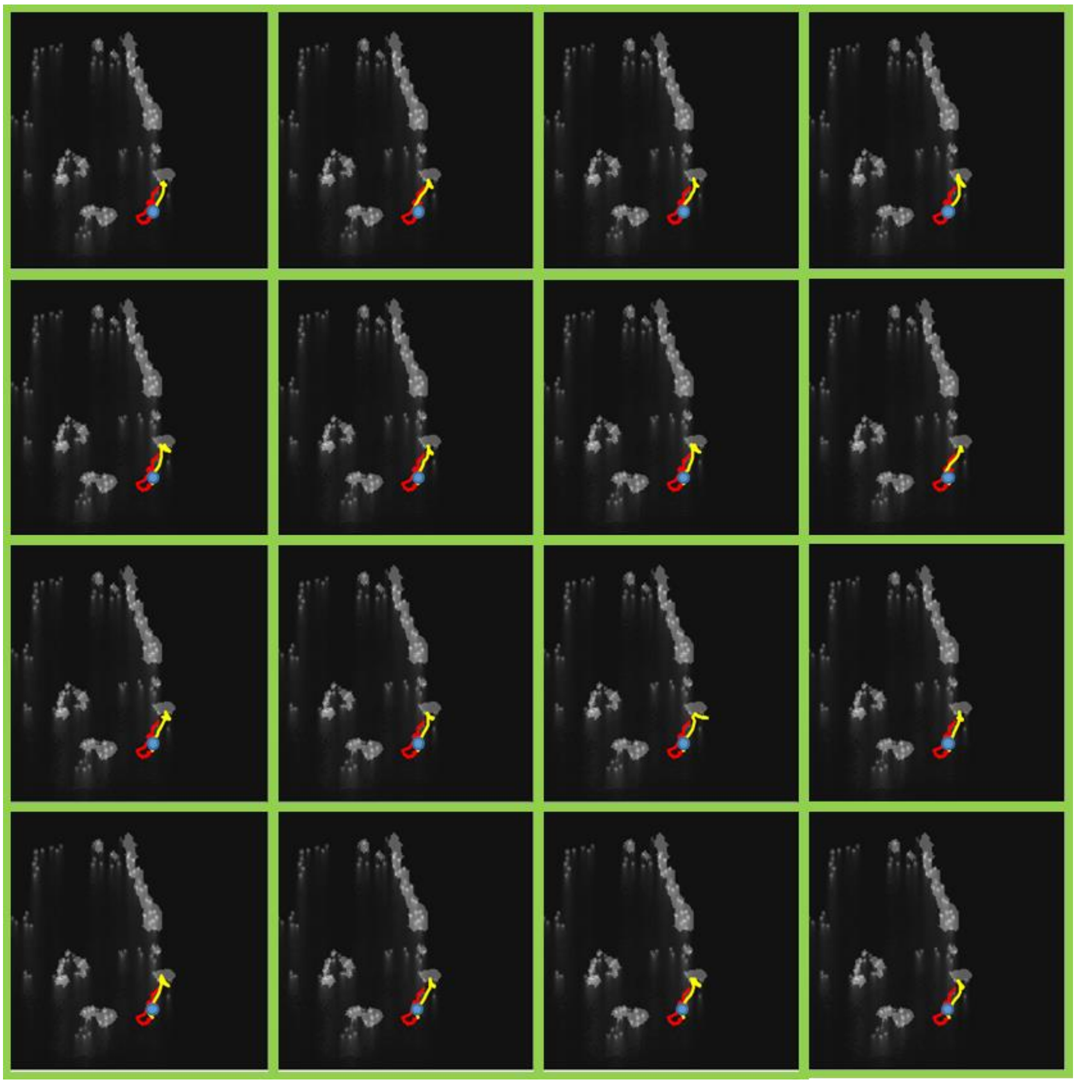

Healthy pediatric donor 2, trajectory 1. 16/16 model runs describe the experimental trajectory.

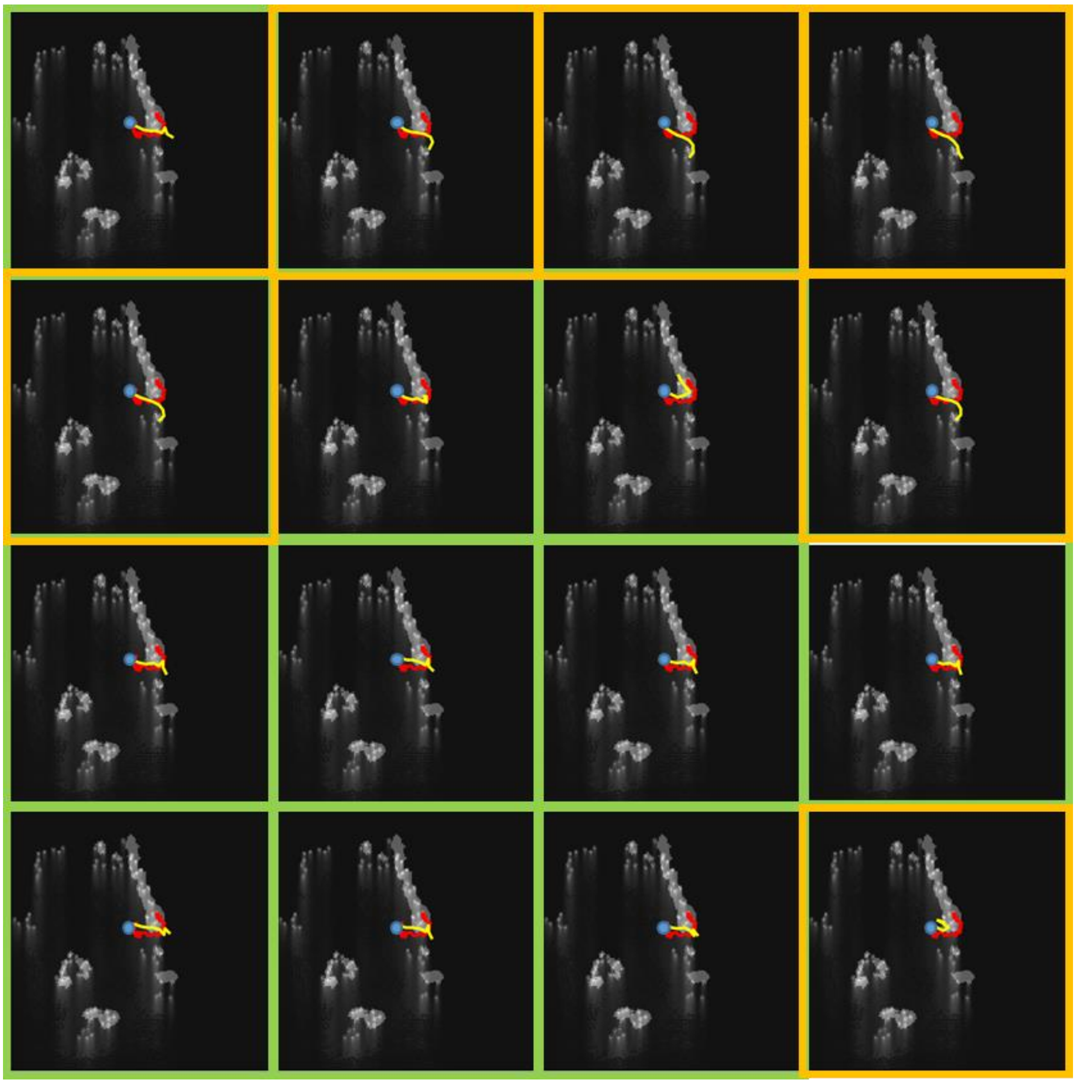

Healthy pediatric donor 2, trajectory 2. 8/16 model runs describe the experimental trajectory.

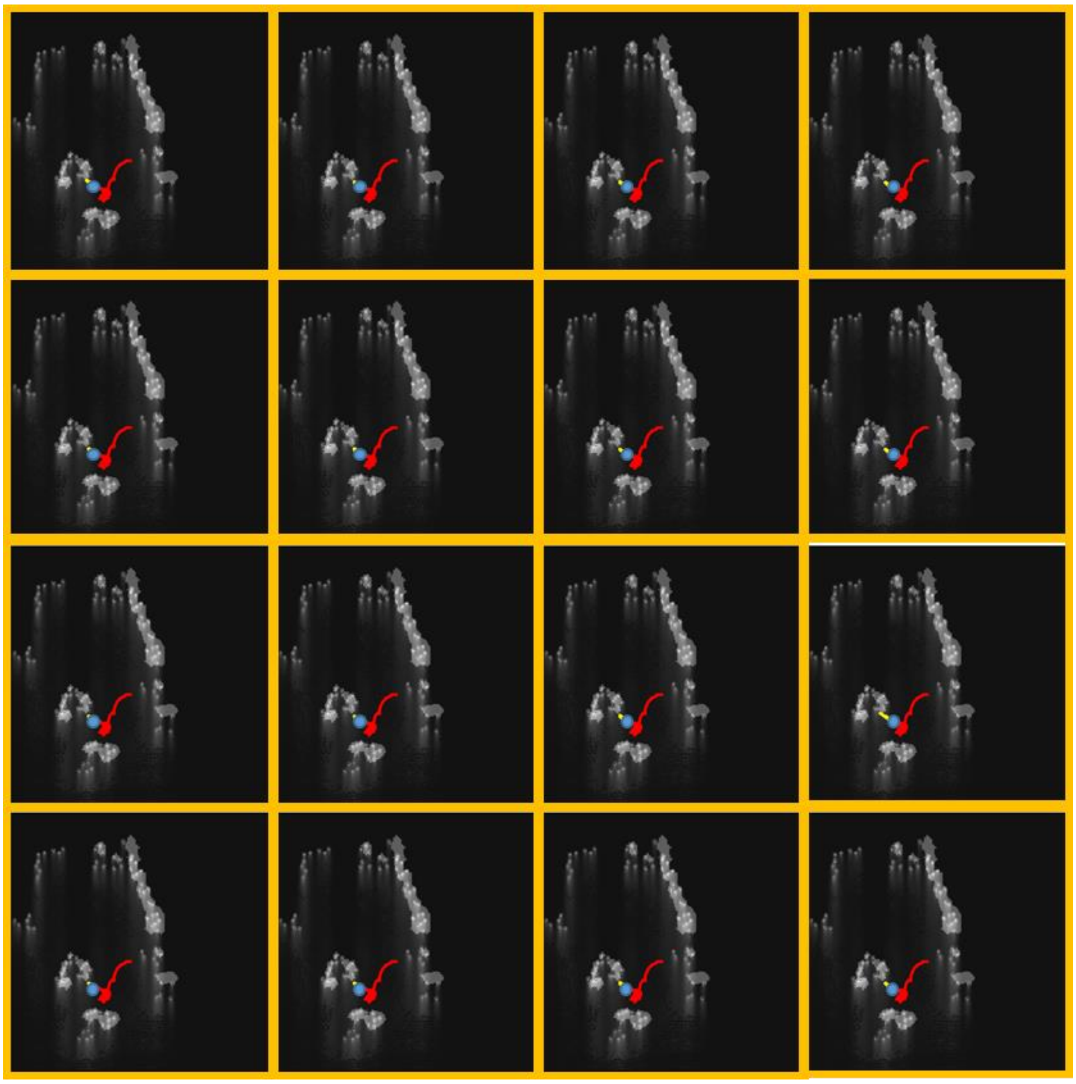

Healthy pediatric donor 2, trajectory 3. 8/16 model runs describe the experimental trajectory.

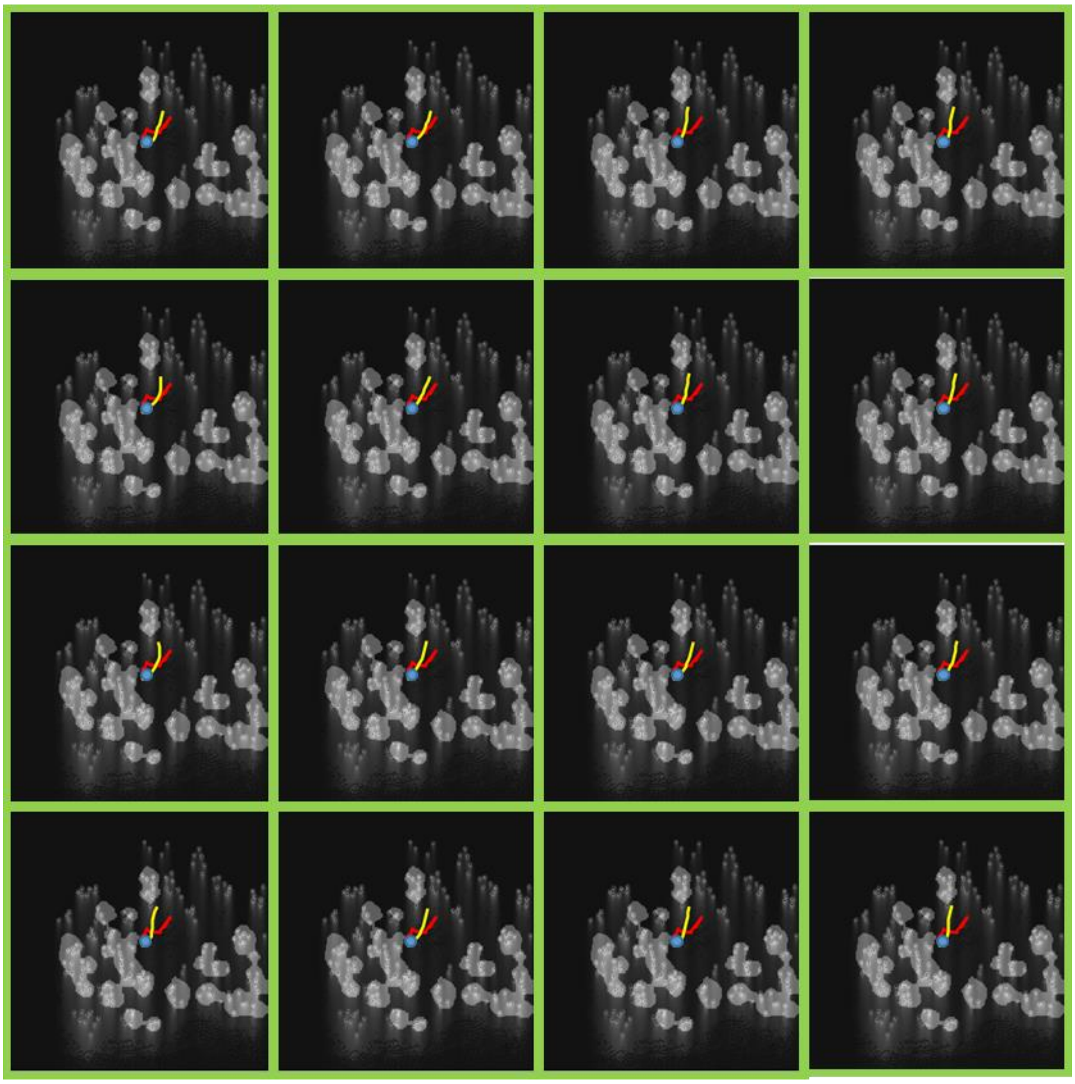

Healthy pediatric donor 3, trajectory 1. 16/16 model runs describe the experimental trajectory.

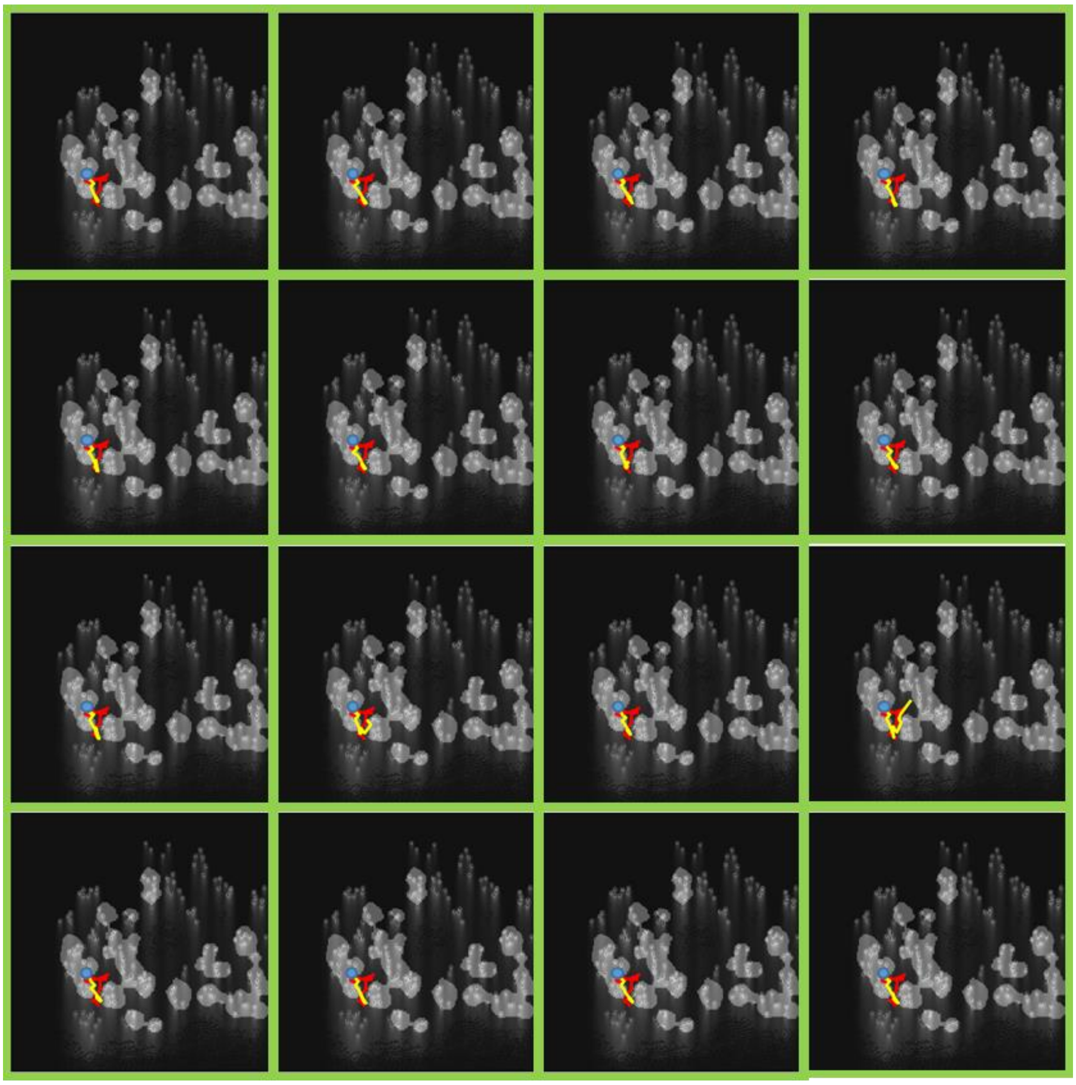

Healthy pediatric donor 3, trajectory 2. 16/16 model runs describe the experimental trajectory.

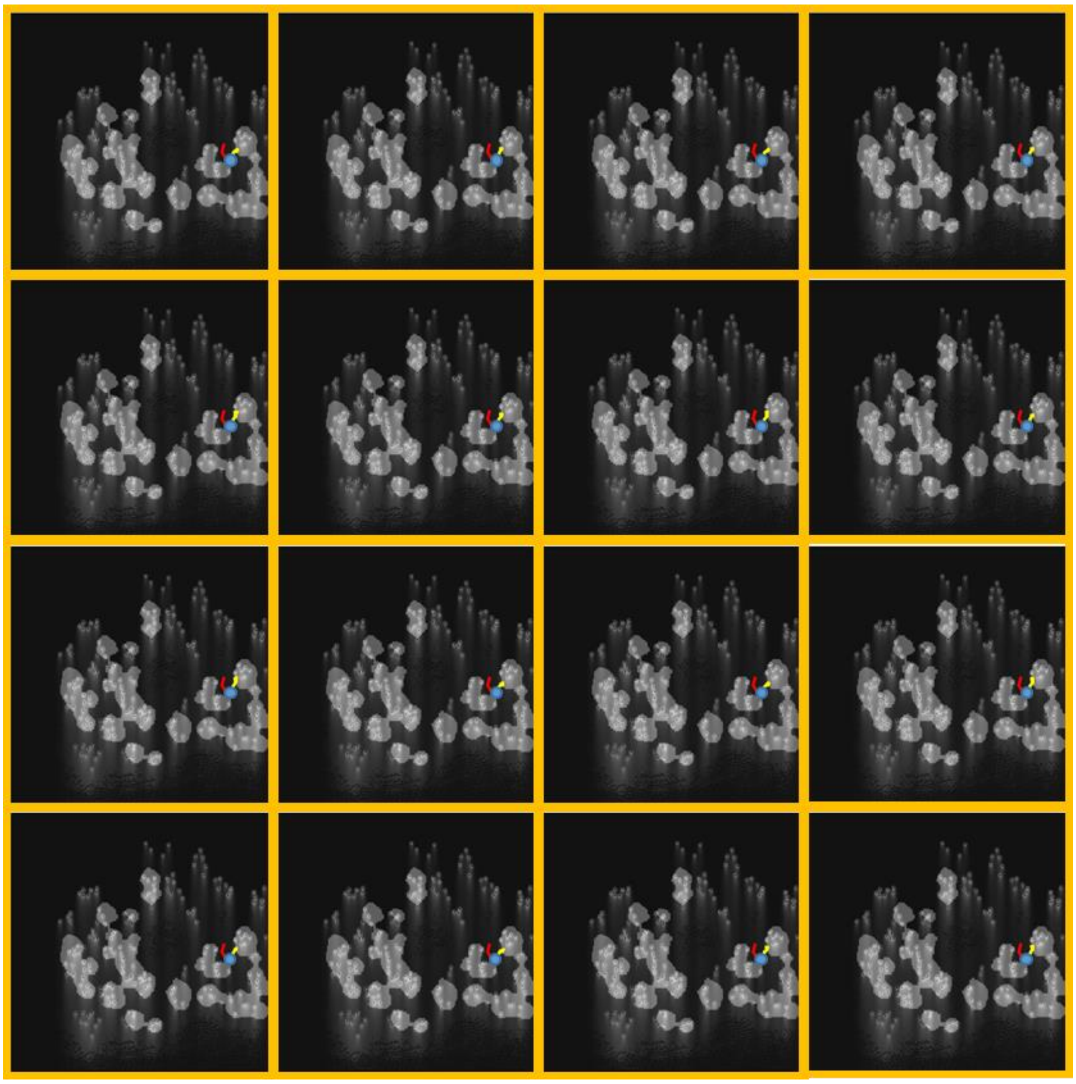

Healthy pediatric donor 3, trajectory 3. 0/16 model runs describe the experimental trajectory.

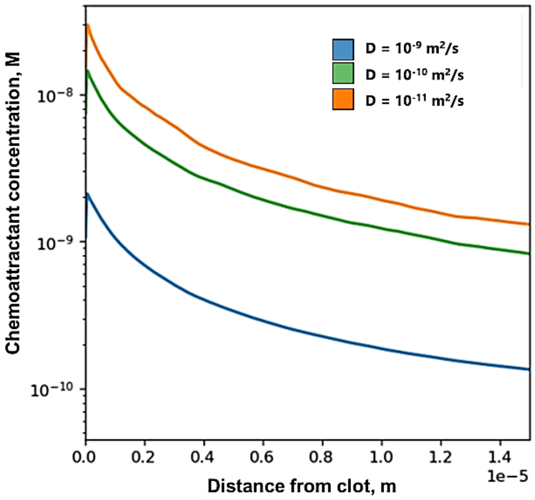

Figure S12a. Model chemokine distribution around thrombi in flow chamber in the same conditions as in Figure 5. The CA diffusion coefficient was varied. CA concentration depends on the diffusion coefficient (D).

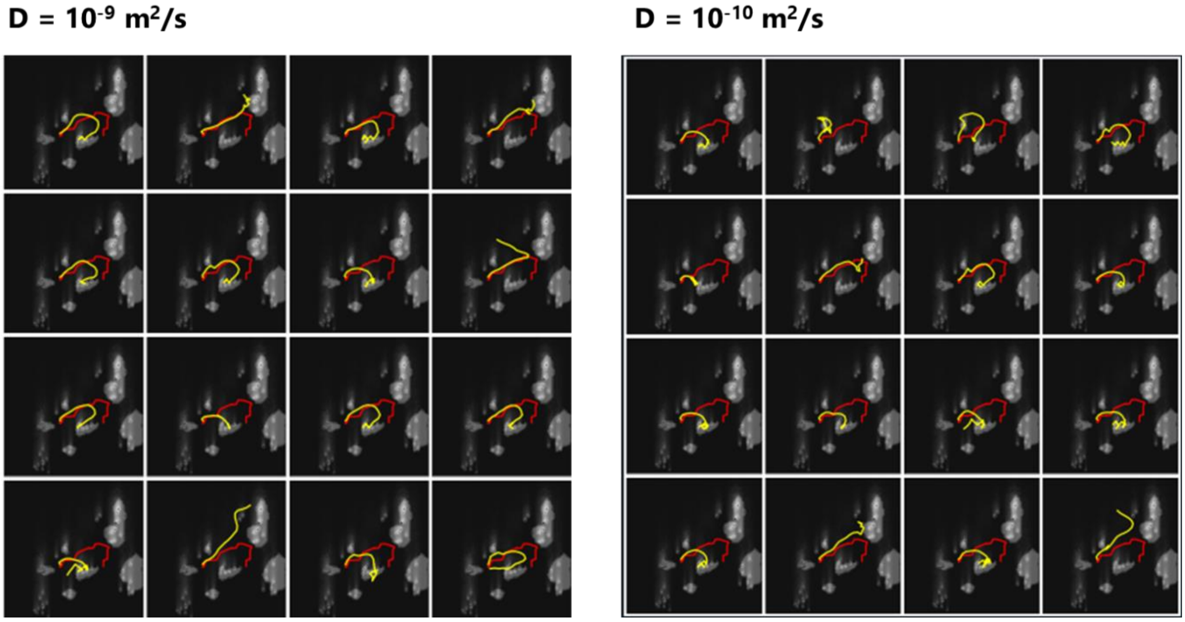

Figure S12b. Individual model runs for two values of the CA diffusion coefficient (D)
